# Global evidence for ultraviolet radiation decreasing COVID-19 growth rates

**DOI:** 10.1073/pnas.2012370118

**Published:** 2020-12-28

**Authors:** Tamma Carleton, Jules Cornetet, Peter Huybers, Kyle C. Meng, Jonathan Proctor

**Affiliations:** ^a^Bren School of Environmental Science and Management, University of California, Santa Barbara, CA 93106-3151;; ^b^Département de Sciences Sociales, École Normale Supérieure Paris-Saclay, 94235 Cachan Cedex, France;; ^c^Department of Earth and Planetary Sciences, Harvard University, Cambridge, MA 02138;; ^d^Department of Economics, University of California, Santa Barbara, CA 93106-3151;; ^e^National Bureau of Economic Research, Cambridge, MA 02138;; ^f^Center for the Environment, Harvard University, Cambridge, MA 02138;; ^g^Data Science Initiative, Harvard University, Cambridge, MA 02138

**Keywords:** COVID-19, ultraviolet radiation, seasonality

## Abstract

With nearly every country combating the 2019 novel coronavirus (COVID-19), there is a need to understand how local environmental conditions may modify transmission. To date, quantifying seasonality of the disease has been limited by scarce data and the difficulty of isolating climatological variables from other drivers of transmission in observational studies. We combine a spatially resolved dataset of confirmed COVID-19 cases, composed of 3,235 regions across 173 countries, with local environmental conditions and a statistical approach developed to quantify causal effects of environmental conditions in observational data settings. We find that ultraviolet (UV) radiation has a statistically significant effect on daily COVID-19 growth rates: a SD increase in UV lowers the daily growth rate of COVID-19 cases by ∼1 percentage point over the subsequent 2.5 wk, relative to an average in-sample growth rate of 13.2%. The time pattern of lagged effects peaks 9 to 11 d after UV exposure, consistent with the combined timescale of incubation, testing, and reporting. Cumulative effects of temperature and humidity are not statistically significant. Simulations illustrate how seasonal changes in UV have influenced regional patterns of COVID-19 growth rates from January to June, indicating that UV has a substantially smaller effect on the spread of the disease than social distancing policies. Furthermore, total COVID-19 seasonality has indeterminate sign for most regions during this period due to uncertain effects of other environmental variables. Our findings indicate UV exposure influences COVID-19 cases, but a comprehensive understanding of seasonality awaits further analysis.

## Significance

There is interest in whether COVID-19 cases respond to environmental conditions. If an effect is present, seasonal changes in local environmental conditions could alter the global spatial pattern of COVID-19 and inform local public health responses. Using a comprehensive global dataset of daily COVID-19 cases and local environmental conditions, we find that increased daily ultraviolet (UV) radiation lowers the cumulative daily growth rate of COVID-19 cases over the subsequent 2.5 wk. Although statistically significant, the implied influence of UV seasonality is modest relative to social distancing policies. Temperature and specific humidity cumulative effects are not statistically significant, and total COVID-19 seasonality remains to be established because of uncertainty in the net effects from seasonally varying environmental variables.

**Fig. 1.**
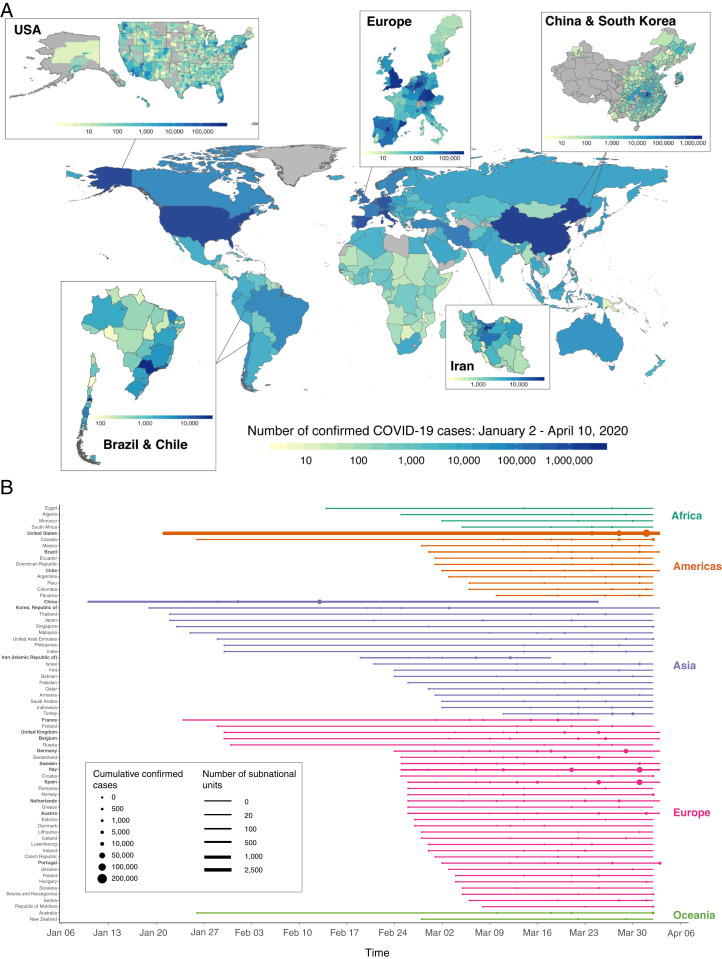
Global assemblage of national and subnational COVID-19 records. (*A*) Total confirmed cases of COVID-19 across 3,235 national and subnational units covering 173 countries from 2 January to 10 April 2020. Darker colors indicate a higher cumulative number of confirmed cases as of 10 April 2020; gray indicates that no data are available. Subnational COVID-19 records were obtained for the United States, Brazil, Chile, Iran, China, South Korea, and 10 European countries. Each box shows within-country heterogeneity in COVID-19 cases for countries with subnational records. (*B*) For the subset of countries with at least 1,000 reported cumulative cases, the period of available COVID-19 data is shown. Data from countries that are in boldface type are available at the subnational level, with the number of administrative units indicated by the thickness of the time series line. Circles indicate the date when cumulative confirmed cases reach specific thresholds, with larger circles indicating higher case counts.

In late 2019, a novel virus species from the family *Coronaviridae*, referred to as severe acute respiratory syndrome coronavirus 2 (SARS-CoV-2), began spreading throughout China (1). Central among SARS-CoV-2 concerns are its relatively high transmissivity and case fatality rates (2). In the ensuing months, the virus spread globally, prompting the World Health Organization to declare a pandemic on March 11, 2020. At the time of this writing, cases of COVID-19, the disease caused by SARS-CoV-2, have been detected in almost every country (Fig. 1*A*), with the number of confirmed global cases in the tens of millions.

Much remains unknown about COVID-19. An important question concerns how environmental conditions modify COVID-19 transmission. In particular, sensitivity to environmental conditions that vary seasonally may allow prediction of transmission characteristics around the globe over the coming months and have implications for seasonal reemergence of infections (3). Prior evidence from a few other viruses suggests the possibility of COVID-19 seasonality. For instance, H3N2, 2009 H1N1, and other strains of influenza exhibit sensitivity to local temperature and specific humidity (4–8). Furthermore, related strains of coronavirus and influenza are inactivated by ultraviolet (UV) radiation (9–11), and a recent laboratory study suggests a similar role whereby UV of a similar spectral distribution to sunlight inactivates SARS-CoV-2 on surfaces (12). The influence of environmental conditions on population-level COVID-19 transmission, however, remains largely unknown (13, 14). Importantly, population-level effects capture human behavioral responses that are typically omitted from laboratory studies.

To estimate the influence of environmental conditions on COVID-19 transmission we first assemble a global dataset of daily confirmed COVID-19 cases. The collated data consist of 1,153,726 COVID-19 cases from 3,235 geospatial units covering 173 countries and five continents (Fig. 1*A* and *SI Appendix*, section B, Tables S2 and S3, and Fig. S1), span 1 January 2020 to 10 April 2020, and have nearly global coverage since March 2020 (Fig. 1*B*). We implement a wide range of data quality control measures, including corrections to the date of reported cases and cross-referencing across multiple sources, to harmonize heterogeneous reporting practices across global sources (*SI Appendix*, section B). For purposes of testing for heterogeneity in response, these case records are also combined with data on location-specific containment policies and testing regimes (15, 16).

We construct our outcome variable as the daily growth rate of confirmed cases, hereafter “daily COVID-19 growth rate,” calculated as the daily change in the logarithm of confirmed cases. Confirmed COVID-19 cases are used because data on recoveries and deaths are not consistently available globally. Growth rates are analyzed because they are a well-established measure for disease spread that reflects changes in transmission characteristics (*SI Appendix*, section A.1). Daily COVID-19 growth rates are assessed in relation to local population-weighted daily temperature, specific humidity, precipitation, and UV from a 0.25° latitude by 0.25° longitude resolution weather reanalysis dataset (17, 18).

We leverage methods from the growing “climate econometrics” literature, which over the last two decades has advanced causal inference techniques to quantify potential impacts of anthropogenic climate change (19–21), including the influence of rising temperatures on civil conflict (22), mortality (23), agricultural yields (24), and human migration (25). The goal of this approach is to mimic controlled experiments by nonparametrically accounting for confounding factors such that the variation in environmental conditions used in the analysis is as good as randomly assigned. Prior work, for example, has used a similar approach to isolate the role of environmental conditions on influenza and provided evidence that low humidity contributes to influenza mortality (26).

**Fig. 2.**
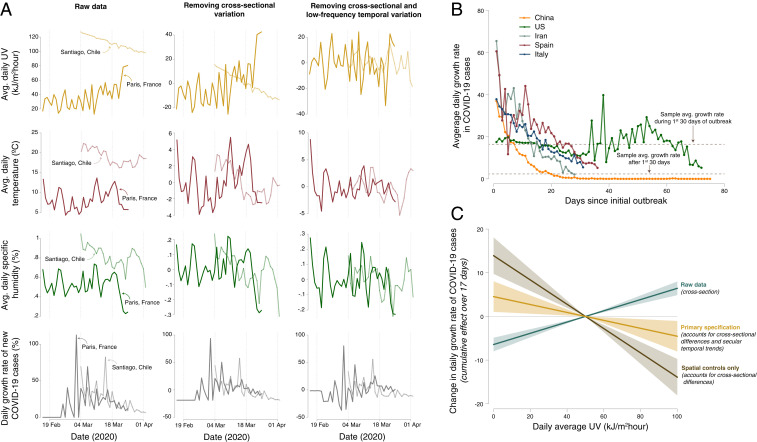
Methodological approach to removing spatial and temporal bias in estimating the impact of environmental conditions on the growth rate of confirmed COVID-19 cases. (*A*) Illustration of, for two example locations, how our empirical strategy isolates idiosyncratic variation in local climatological conditions through the inclusion of semiparametric controls (i.e., “fixed effects”) in a panel regression framework (*SI Appendix*, Eq. **S1**). *A*, *Left* displays raw time series data from Paris, France (dark color) and Santiago, Chile (light color) for UV exposure (gold), temperature (maroon), specific humidity (green), and daily COVID-19 growth rates (gray). *A*, *Center* displays these same time series, after location-specific fixed effects have been removed. *A*, *Right* shows the residual variation used in empirical estimation; location-specific averages, day-of-year averages, and country-specific weekly temporal variation are removed using a suite of fixed effects described in *SI Appendix*, section A.2. The resulting time series no longer display average differences across space or trending behavior within a location, thus removing the possibility that unobserved time-constant or trending variables may confound empirical estimates. (*B*) Average daily growth rates in confirmed COVID-19 cases for five select countries, indexed against the number of days since the first case was detected. Values shown are unweighted average growth rates computed across all subnational units within each country (Fig. 1). Note that increased variance in the United States average growth rate after approximately 30 d since initial outbreak occurs due to a limited sample of counties for which confirmed cases have been reported for greater than 30 d. (*C*) Estimates of the cumulative effect of UV exposure on subsequent daily COVID-19 growth rates from three variations of the regression in *SI Appendix*, Eq. **S1**. Lines indicate the effect of changing daily average UV from the sample average of 50 $kJ\cdot m^{-2}\cdot h^{-1}$ to a given value; shading shows 95% confidence intervals. In gold is the primary specification used throughout our analysis, which includes the full set of semiparametric controls described in *SI Appendix*, section A.2. In teal, all spatial and temporal controls are removed from the estimation (i.e., data shown in *A*, *Left* are used), introducing confounding variation across space and time and leading to substantial bias in the estimated effect of UV on growth rates. In brown, location-specific fixed effects are included, while temporal controls are omitted (i.e., data shown in *A*, *Center* are used), introducing confounding low-frequency temporal variation and again leading to a biased estimator.

Although a strictly causal interpretation of results is not possible in any observational study, our research design (detailed in *SI Appendix*, section A.2) addresses four key issues associated with prior observational analyses. First, a location’s prevailing social and economic characteristics, which likely influence COVID-19 transmission, may also be correlated with its average environmental conditions. For example, countries that are cooler on average tend to have higher income per capita (27), with the latter feature associated with more widespread access to medical care, testing, and reporting. Indeed, a recent review by the National Academies of Sciences, Engineering, and Medicine notes that temperature and humidity effects on COVID-19 remain inconclusive in part because of these cross-sectional differences (13). We address this concern through the use of location-specific “fixed effects,” or dummy variables, which flexibly control for all differences in time-invariant social and economic characteristics and data quality across geospatial units (28). Fig. 2*A*, *Left* and *Center* illustrates how this spatial demeaning affects our data for two sample locations—Santiago, Chile and Paris, France—where average climatological conditions differ strongly and where the timing and intensity of the disease evolved distinctly. Empirical estimation relying on the data shown in Fig. 2*A*, *Left* would conflate differences in environmental conditions across these two locations with the many other differences between these cities, such as baseline population density or health (Fig. 2*C*, discussed in *Results*).

Second, within any given location, there are temporal trends in both daily environmental conditions and the COVID-19 growth rate, with the latter due to anticontagion policies and inherent dynamics of transmission that are unrelated to environmental conditions (*SI Appendix*, section A.1). We address the concern that such trends may bias causal estimates through the inclusion of flexible location-specific temporal controls that remove low-frequency temporal variation in both COVID-19 and environmental conditions. We additionally employ global-scale, day-of-sample controls to account for any high-frequency common shocks to the evolution of the disease or its reporting across the globe. The resulting location-specific, high-frequency fluctuations in environmental conditions after removal of trends appear to exhibit quasi-random variation (19–21), as illustrated in Fig. 2*A*, *Right*.

Third, a number of different environmental variables have been postulated to affect transmission, including UV, temperature, humidity, and precipitation (29–33). These atmospheric variables are dynamically linked (*SI Appendix*, Fig. S3). For instance, solar radiation is correlated with relative humidity and precipitation through cloud formation and convection. Such associations confound causal estimates if key variables are omitted from the analysis (34). We address this concern by simultaneously estimating the effects of UV, temperature, humidity, and precipitation, such that the effect of any single environmental variable is estimated after accounting for correlations with other specified environmental variables.

Fourth, any modification of transmission will appear with some delay in observations of confirmed COVID-19 cases. The length of this delay between transmission and case confirmation includes the incubation period as well as time required to diagnose the disease. Prior case studies have identified the incubation period to range between 4 and 7 d (35–37) and the period between symptom onset and case confirmation to range between 2 and 7 d (38), implying a combined delay of 6 to 14 d between transmission and case confirmation. In a population-level study like ours, where individuals reside in diverse testing and reporting regimes, we expect there to be heterogeneity in lag lengths across different individuals and regions of the world. Because the distribution of delays across a population is unknown, estimation of a population-level causal response requires a statistical approach that accounts for the pattern of lagged effects in a data-driven manner. To this end, we employ a temporal distributed lag regression model that enables flexible, data-driven estimates of the effects of environmental conditions on the COVID-19 growth rate up to 2.5 wk later, a period long enough to incorporate the range of delays detected by prior studies. To quantify the total effect of environmental exposure, we sum the estimated effects across all lags for each variable (21, 39). As is standard in investigations of dynamic effects of the environment on socioeconomic conditions (40–45), we treat this “cumulative effect” as our main statistic of interest.

Together, inclusion of these four elements in a panel regression model allows us to quantify the impact of quasi-random daily variations in environmental conditions on the subsequent evolution of the COVID-19 caseload (*SI Appendix*, section A.2 and Eq. **S1**). We examine the sensitivity of our conclusions to a range of alternative statistical model formulations that, among other things, vary the stringency of the spatiotemporal controls and additionally control for the local timing of COVID-19 outbreaks, testing regimes, and COVID-19 containment policies. The ability of our statistical approach to recover the effects of environmentally driven changes in transmission on the COVID-19 growth rate is confirmed by applying our statistical model to synthetic data simulated by a standard susceptible–exposed–infected–recovered (SEIR) model with an environmentally perturbed transmission parameter (46), as detailed in *SI Appendix*, section A.1 and Figs. S12 and S13.

Finally, we note that several elements of our statistical approach also address concerns regarding systematic reporting biases with COVID-19 case data. First, our use of the growth rate of COVID-19 cases as the outcome variable accounts for location-specific reporting biases in the level of COVID-19 cases. Second, time-invariant reporting biases in COVID-19 growth rates are removed by location-specific fixed effects. Third, inclusion of flexible country-specific time trends accounts for time-varying differences in reporting bias across countries. Fourth, we address remaining differences due to testing regimes by demonstrating that our main result is invariant to controlling for country-level testing policy over time. Remaining challenges associated with identification of environmental effects on COVID-19 transmission are considered in *Discussion*.

## Results

On average across our sample, confirmed COVID-19 cases grow at a rate of 13.2%/d (SD of 24.4%), equivalent to a doubling time of 5.2 d. Growth rates generally decreased over the first months of the outbreak, with the sample average growth rate falling from 15.7% during the first month of a location’s outbreak to 2.3% in subsequent months (Fig. 2*B*). These declines are consistent with the epidemiological dynamics of the virus (*SI Appendix*, section A.1) and with strengthened containment efforts over time, although they may also reflect other factors.

**Fig. 3.**
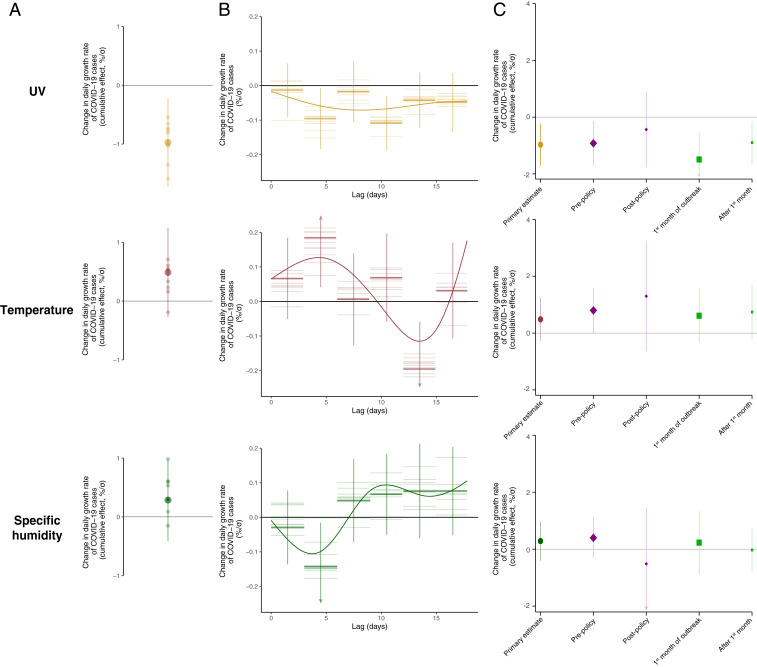
Empirical estimates of the relationship between COVID-19 and local environmental conditions. (*A*) The estimated cumulative effect of a change in daily UV, temperature, or specific humidity on subsequent daily COVID-19 growth rates over the following 17 d. All estimates are standardized, such that effect sizes represent percentage point changes per SD (σ). Our central estimate (*SI Appendix*, Eq. **S1**) is shown by the large circle, along with vertical lines representing the 95% confidence interval, which is calculated allowing for arbitrary correlation of model errors within administrative units over time. Smaller circles show estimates of the cumulative effect from alternative plausible statistical model formulations that, among other things, vary the stringency of the spatiotemporal controls or additionally control for the local timing of COVID-19 outbreaks, testing regimes, or COVID-19 containment policies (*SI Appendix*, Table S1, cols. 1–7 and Figs. S5–S7). (*B*) The temporal dynamics of each estimated lagged effect of UV, temperature, and specific humidity on the daily COVID-19 growth rate over 17 d for our central estimate (thick lines, one for each coefficient in *SI Appendix*, Eq. **S1**) and alternative model formulations (thin lines, same alternative models as in *A*). Coefficients have been divided by three to show per-day effects. The displayed curve is a fit to the estimated lag coefficients from our central estimate (*SI Appendix*, section A.3). *C* replicates the cumulative effect of each weather variable on daily COVID-19 growth rates from the primary specification in *A* in gold (UV), maroon (temperature), and green (specific humidity). In purple, treatment effects are reported for the period before an administrative unit imposed any social distancing measures (large purple diamond) and after such measures were put in place (small purple diamond). Similarly, in light green, treatment effects of each weather variable are reported for the first 30 d of the location-specific outbreak (large green square) and for all dates after the first 30 d (small green square). Vertical lines indicate 95% confidence intervals. Arrows indicate where confidence intervals have been truncated for display. Effects of social distancing policies and outbreak duration on individual lag coefficients for all three weather variables are shown in *SI Appendix*, Fig. S8.

Applying a panel-regression model to growth rates (*Materials and Methods* and *SI Appendix*, section A.2) demonstrates a statistically significant effect whereby increases in surface UV intensity lower subsequent COVID-19 growth rates. In our primary specification, we estimate that a 1 $kJ\cdot m^{-2}\cdot h^{-1}$ increase in local UV reduces local COVID-19 growth rates by 0.09 (±0.04, P=0.01, range indicates ± 1 SD) percentage points over the ensuing 17 d (Fig. 3*A* and column (col.) 3 of *SI Appendix*, Table S1). The effects associated with UV are consistently negative across lags and peak in magnitude after 9 to 11 d (Fig. 3*B* and *SI Appendix*, Fig. S5). This delay between UV exposure and changes in the COVID-19 growth rate is consistent with the reported time frame between exposure to the virus and its detection (36, 47, 48). The estimated UV effects imply that a sample SD increase in UV (10.73 $kJ\cdot m^{-2}\cdot h^{-1}$, accounting for semiparametric controls) reduces growth by 0.97 (±0.38) percentage points or 7% of the sample average growth rate of 13.2%. This amounts to an increase in doubling time of COVID-19 cases from 5.2 d—at the average growth rate—to 5.7 d (±0.2). In contrast, the effects of higher temperatures and higher levels of specific humidity are of less consistent sign, with cumulative effects over the 17-d interval being statistically insignificant and of opposite sign to that of the lag with the greatest magnitude (Fig. 3 *A* and *B* and *SI Appendix*, Table S1). A SD increase in temperature (2.84 °C) and specific humidity (0.13%) leads to small and uncertain estimated effects on the growth rate of 0.49 and 0.29 percentage points, respectively.

The effect of UV radiation on the COVID-19 growth rate (Fig. 3 *A* and *B*) is robust to a range of alternative statistical models, including controls for days since initial outbreak of the virus in each location (col. 1 in *SI Appendix*, Table S1); linear, country-specific time trends (col. 2 in *SI Appendix*, Table S1); controls for future weather as a test of reverse causality (col. 4 in *SI Appendix*, Table S1); controls for temporally and spatially varying policies, such as work-from-home policies, school closures, and event cancellations (col. 5 in *SI Appendix*, Table S1 and col. 2 in *SI Appendix*, Fig. S5); controls for the extent of COVID-19 testing availability (col. 6 in *SI Appendix*, Table S1); semiparametric controls that allow for within-country differential seasonality, in addition to country-wide seasonal patterns (col. 7 in *SI Appendix*, Table S1 and col. 3 in *SI Appendix*, Fig. S5); removal of country-level data and addition of semiparametric controls that allow for country-by-day specific shocks (col. 8 in *SI Appendix*, Table S1 and col. 4 in *SI Appendix*, Fig. S5); analysis using a Poisson regression model (*SI Appendix*, Eq. **S2**) in place of ordinary least squares (col. 9 in *SI Appendix*, Table S1 and col. 5 in *SI Appendix*, Fig. S5); replacing specific humidity with relative humidity (*SI Appendix*, Fig. S6); and changing the number of lags included in the estimating equation (*SI Appendix*, Fig. S7). We further show our estimates are insensitive to outliers using a procedure whereby we reestimate our cumulative effect after systematically dropping each of our 3,235 geospatial units (*SI Appendix*, Fig. S9). Finally, we estimate an alternative model that allows for nonlinearities between weather conditions and COVID-19 growth rates and find that the UV effect exhibits strong linearity (*SI Appendix*, Fig. S10 and Eqs. **S3**–**S4**). Whereas the significance and magnitude of the cumulative UV effect are stable across the different model specifications, the cumulative effects of temperature and humidity are insignificant across all model specifications and have inconsistent sign (Fig. 3*A* and *SI Appendix*, Table S1).

In contrast to UV estimates being insensitive to the addition or modification of controls, omitting location and time fixed effects or omitting temporal trends leads to substantially biased estimates of the environmental determinants of transmission compared to our primary specification. In Fig. 2*C* we show three sets of response functions relating daily average UV exposure to its cumulative effect on subsequent daily COVID-19 growth rates, where responses are centered relative to the in-sample average UV value of 50 $kJ\cdot m^{-2}\cdot h^{-1}$. When all semiparametric controls are omitted (teal line in Fig. 2*C*, corresponding to data shown in Fig. 2A, *Left*), the estimated effect of UV is of the opposite sign to that hypothesized in prior literature and estimated in our primary specification (gold line in Fig. 2*C*, corresponding to data shown in Fig. 2*A*, *Right*). Similarly, omission of temporal controls (brown line in Fig. 2*C*, corresponding to data shown in Fig. 2*A*, *Center*) overestimates the negative effect of UV on COVID-19 growth rates, as time-trending unobservable factors, such as changes in reporting and the general progression of the disease, correlate with gradual changes in UV exposure. These results highlight the empirical importance of adequately removing the influence of key confounding factors that have to date limited the ability to determine whether and how environmental conditions constrain the evolution of COVID-19 (13, 14).

The cumulative lagged effect of weather conditions on COVID-19 growth rates reflects the average treatment effect over all geospatial units and over the course of the observed pandemic (Fig. 3*A*). It can be inferred, however, that effective social distancing policies will reduce any relationship between UV exposure and transmission of COVID-19. Transmission must be nonnegative; thus, any single reduction limits the scope for further reduction. Consistent with this, we find suggestive evidence that social distancing policies such as school closures, mandatory work from home orders, and large event cancellation regulations weaken the link between COVID-19 and weather conditions. Specifically, using a binary policy variable indicating whether an administrative unit has any one of a set of social distancing measures in place (*SI Appendix*, section B.3), we find that the cumulative effect of 1 $kJ\cdot m^{-2}\cdot h^{-1}$ increase in UV falls from −0.09 (±0.04, P = 0.02) before policies are introduced to −0.04 (±0.06, P = 0.52) after policies are put in place (Fig. 3*C*).

Similarly, the effect of UV exposure on transmission of COVID-19 is likely to decline over the course of the pandemic, as social distancing policies are enacted and individuals gain more awareness of and information about the virus. Indeed, we find suggestive evidence that the cumulative effect of UV on the growth rate of COVID-19 cases is stronger during the first month of a population’s outbreak (−0.14 ± 0.04) than in subsequent months (−0.08 ± 0.04). The pattern of effect attenuation shown in Fig. 3*C* is also observed for the individual lags of temperature and specific humidity that have the largest magnitude (*SI Appendix*, Fig. S8), although cumulative effects of temperature and specific humidity are statistically indistinguishable from zero both with and without public health policies in place (Fig. 3*C*).

**Fig. 4.**
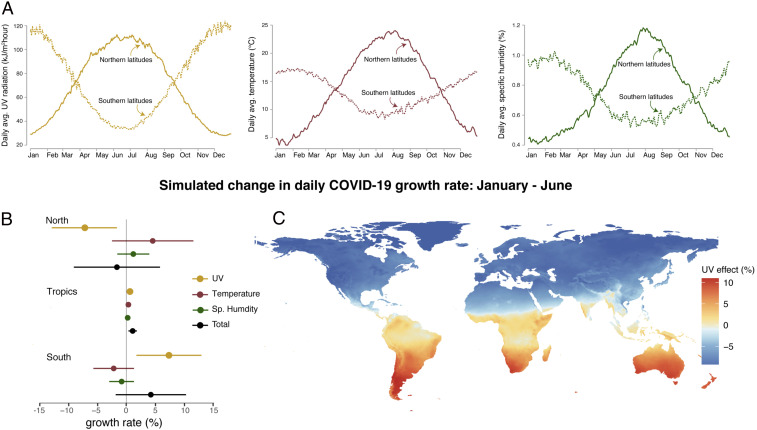
Seasonality in the simulated COVID-19 growth rate. (*A*) The average seasonal cycle of UV, temperature, and specific humidity for northern (above 23° north) and southern (below 23° south) latitudes. (*B*) The individual impacts of seasonal changes in UV (gold), temperature (maroon), and specific humidity (green), as well as their combined effect (black), from January to June. Points indicate average simulated impacts for northern latitudes, the tropics (23° south to 23° north), and southern latitudes. Horizontal lines show 95% confidence intervals, which account for uncertainty in statistical parameters. (*C*) Map of the influence of expected seasonal changes in UV alone on the COVID-19 growth rate from January to June.

The estimated effect of UV on the COVID-19 growth rate has seasonal implications (Fig. 4). To illustrate the role of changing UV in the evolution of the disease over the year, we use the cumulative effect of UV recovered in Fig. 3*A*, along with the local seasonal climatology of surface UV intensity between January and June, to quantify how seasonality in UV altered daily COVID-19 growth rates across the world (*SI Appendix*, section A.4). This period, besides encompassing our entire data period, also covers the full range of seasonal UV exposure experienced in any location, as shown in Fig. 4*A*. Between January and June, the increase in seasonal UV exposure ($+81.0\;kJ\cdot m^{-2}\cdot h^{-1}$) lowered extratropical Northern Hemisphere (above 23° north) COVID-19 growth rates by 7.4 (±2.9) percentage points. Over the same period, the seasonal decline in UV ($-80.7\;kJ\cdot m^{-2}\cdot h^{-1}$) raised growth rates by 7.3 (±2.9) percentage points in the extratropical Southern Hemisphere (below 23° south) (Fig. 4 *B* and *C*). This seasonal change amounts to an increase in the doubling time from an average of 5.2 to 11.8 d in the extratropical Northern Hemisphere and a corresponding decrease to 3.4 d in the extratropical Southern Hemisphere. Seasonality in UV in the coming boreal winter reverses this pattern. Between June and December, our estimates imply that COVID-19 growth rates increase by 7.8 (±3.0) percentage points in the extratropical Northern Hemisphere and fall by 7.7 (±3.0) percentage points in the extratropical Southern Hemisphere from changes in UV (*SI Appendix*, Fig. S11). These changes in COVID-19 growth correspond to lowering the average doubling time to 3.3 d in northern latitudes and raising it to 12.6 d in southern latitudes.

As a whole, the tropics display moderate seasonal changes driven by UV, with our simulations generating an increase of 0.57 (±0.22) percentage points between January and June due to an decrease in UV exposure of $6.3\;kJ\cdot m^{-2}\cdot h^{-1}$. A notable regional exception is that the onset of the South Asian monsoon causes decreased surface UV regionally in June, thus raising summer COVID-19 risks. We emphasize that these simulations are merely illustrations of the potential seasonal influence of UV. Changes in population immunity rates, genetic mutations of the virus, and public health policies, among many other factors, could alter the sensitivity of COVID-19 to environmental conditions, causing future seasonal implications to differ from those derived over our sample period.

Other seasonally varying climate variables may have also influenced COVID-19 cases during the first 6 mo of infection, including temperature and specific humidity (Fig. 4*A*), whose effects on COVID-19 transmission rates remain uncertain. Indeed, similar exercises for northern and southern latitudes using only January to June seasonality in temperature or specific humidity do not yield changes in daily COVID-19 growth rates during these first 6 mo that are statistically distinguishable from zero because the cumulative effect of each variable is uncertain (maroon and green bars in Fig. 4*B*). The uncertain contribution of these variables renders the total effect of seasonality across all three variables uncertain, with 95% confidence intervals for the cumulative effect containing zero for northern and southern latitudes (black bars in Fig. 4*B*). In the tropics, seasonality is smaller and more complex but the total effect is significant between January to June because UV, temperature, and specific humidity influences align.

## Discussion

Using a global, harmonized dataset of daily COVID-19 cases, we find that the daily growth rate of confirmed COVID-19 cases responds negatively to increased UV. Importantly, variations in the COVID-19 growth rate lag variations in UV by up to 2.5 wk, consistent with times required for incubation, testing, and reporting. The UV response is robust to a range of model specifications and controls. However, the influence of other seasonally varying environmental conditions is not precisely estimated. We hope that the distributed-lag, panel-regression framework employed here may underlie further analyses of the influence of environmental conditions on COVID-19 transmission, particularly as COVID-19 data availability improves.

Our findings are consistent with the hypothesis that UV radiation alters COVID-19 transmission rates by more rapidly deactivating the SARS-CoV-2 virus residing on surfaces or in aerosol form, as suggested by recent laboratory studies (12). We cannot rule out, however, that UV may also influence the incubation period of SARS-CoV-2, testing rates, behavior such as time spent indoors or socializing (49), or other disease-transmission or monitoring properties. Our findings also indicate that climate has a modest effect on viral transmission relative to social distancing. Measurements of the effect of social distancing policies on COVID-19 growth early in the epidemic (16) are 3 to 6 times larger than the influence of UV seasonality that we estimate for the extratropical Southern and Northern Hemispheres. Due to minimal seasonality in the tropics, social distancing policy effect sizes are 35 to 85 times larger than the effect of UV seasonality on COVID-19 growth that we estimate in this region (*SI Appendix*, section A.4). Because factors such as social distancing policies have a larger influence than seasonal variation in UV, and high susceptibility to date among the global population permits for rapid transmission, COVID-19 growth is unlikely to exhibit substantial seasonality, at least in the near term (50, 51). If COVID-19 becomes widely established, environmental influences may become more important for inducing seasonal variations in the growth of infections (50).

Our study has a number of important limitations. First, as is true in any empirical study of disease, we can only observe cases that are confirmed. The fact that confirmed cases of COVID-19 are likely well below the actual number of infections (52) would not, of itself, affect estimates of the growth rate if confirmed cases were a constant ratio of the actual value. However, other factors such as variations over time in the rates of testing or testing procedures (53, 54) could alter observed growth rates. In some settings, the bias in growth rates due to such time-varying underreporting has been found to be quantitatively small (16). Moreover, our research design ensures that such imperfect reporting does not systematically bias our estimated effects of environmental conditions, provided that testing procedures or reporting practices are uncorrelated with climatological variables (*SI Appendix*, section A.2). We additionally address this concern statistically by accounting for location-specific trends in confirmed COVID-19 cases and by controlling for the availability of COVID-19 testing at the country level (*SI Appendix*, section B and Table S1), although reporting issues could remain.

Second, it is possible that the behavioral response to random day-to-day fluctuations in UV (and other environmental variables) differs from the behavioral response to expected seasonal changes. For example, an anomalously sunny day in March may elicit different human behavior than a day in July with the same UV exposure. It may be possible to estimate such state-dependent effects after the accrual of multiple seasons of data. There is also a potential concern that the slow response of the dynamic system of the disease would damp the amplitude of the response to high-frequency day-to-day environmental fluctuations. We find using stochastic simulations of the SEIR model, however, that simulated UV-induced variations in transmission are generally insensitive to the frequency of perturbations (*SI Appendix*, section A.1 and Fig. S13*G*).

Third, some studies suggest a relationship between air pollution and COVID-19 transmission and mortality (55–58). Although it is theoretically possible that the negative effect of UV that we recover is partially explained by air pollution attenuating UV and increasing COVID transmission, this is unlikely to be the case, given that day-to-day variation in UV is driven primarily by changes in cloud cover, with much smaller contributions coming from variations in ozone, aerosols, and water vapor (59).

Finally, although we know of only one publicly available laboratory study of the UV–COVID-19 transmission relationship (12), we view our approach as complementary. Although laboratory studies isolate the biology of virus transmission, our statistical approach using observed COVID-19 cases captures those channels as well as behavioral adjustments individuals make in response to short-term UV fluctuations, such as decisions to spend time indoors or outside, to exercise, or to attend social gatherings, and other activities and health investments (49, 60). As public health officials grapple with the costs and benefits of a range of possible responses to the current pandemic, quantifying the influence of both biologically direct and behaviorally induced modification channels is essential to building appropriate policies.

## Materials and Methods

To construct a harmonized global dataset of geolocated daily confirmed COVID-19 cases, we assemble publicly available data from national governments, subnational authorities, and newspapers. Subnational sources are described in *SI Appendix*, Table S2; for all countries for which subnational records were not publicly available at the time of writing, we use national-level records provided by the Johns Hopkins University Center for Systems Science and Engineering (61). No statistical methods were used to predetermine sample size.

We link COVID-19 case data to gridded daily weather data from the European Centre for Medium-Range Weather Forecasts Reanalysis 5th (ERA5) product (17) by calculating the population-weighted average UV, temperature, specific humidity, and precipitation for each day across all grid cells within each administrative unit (*SI Appendix*, section B). We additionally combine case records with data on location-specific containment policies and testing regimes from refs. (15, 16).

UV radiation is represented as including wavelengths from 200 to 440 nm. Limitations associated with the representation of radiative transfer associated with ERA5 reanalysis prevent us from distinguishing between UVa (400 to 315 nm), UVb (315 to 280 nm), and UVc (280 to 100 nm) effects. Higher-energy UVc and UVb radiation may more rapidly deactivate SARS-CoV-2 than UVa but is also more readily absorbed in the atmosphere (12, 62). A more detailed analysis of the transmission and reflection of different types of UV in association with destruction of the SARS-CoV-2 is a fruitful area for future research.

We statistically estimate the effect of weather on the daily growth rate of confirmed COVID-19 cases using a longitudinal (i.e., panel) regression model. Daily COVID-19 growth rates are estimated as a linear function of UV, temperature, specific humidity, and precipitation exposure over the preceding 17 d in a model allowing the effect of each environmental variable to differ across lag intervals of 3 d (*SI Appendix*, Eq. **S1**). We include indicator variables (i.e., fixed effects) for each subnational or national administrative unit, for each day of the sample, and for each country by week in the sample. We calculate standard errors accounting for serial correlation across days within each administrative unit. Clustering at the country level, which further allows for spatial correlation of arbitrary form within administrative units from the same country, does not discernibly change the precision of our estimates. All *P* values are calculated using two-sided tests. We test for heterogeneity in the estimated effect of weather conditions on COVID-19 growth rates by interacting the lagged weather variables with binary variables indicating whether containment policies are in place and whether the observation is in the first month of the outbreak (*SI Appendix*, section A.2).

Seasonal simulations (Fig. 4) use the daily seasonal climatology of UV, temperature, and specific humidity, which we calculate by averaging daily data from the ERA5 reanalysis product over the years 2015 to 2019 (*SI Appendix*, section A.4).

Throughout the analysis daily growth rates of confirmed COVID-19 cases are shown using units of percent. For example, a growth rate of 0.13 is approximately equivalent to a growth rate of 13%. Changes in growth rates are given in units of percentage points. For example, a reduction of the growth rate by 1 percentage point would change the growth rate from 13% to 12%.

## Supplementary Material

Supplementary File

## Data Availability

All data used in this analysis are compiled from free, publicly available sources. Code and data used in the analysis are publicly accessible in Zenodo (DOI: 10.5281.zenodo.3829621).

## Change History

February 3, 2021: The data availability section has been updated.
