# Supplementary material for: Global evidence for ultraviolet radiation decreasing COVID-19 growth rates
Source: Proc Natl Acad Sci U S A. 2020 Dec 28;118(1):e2012370118. doi: 10.1073/pnas.2012370118 (PMC7817154; doi:10.1073/pnas.2012370118)
Supplement: Supplementary File [file pnas.2012370118.sapp.pdf]

Supporting Information (SI) Appendix

Global evidence for ultraviolet radiation decreasing COVID-19 growth rates

Tamma Carleton[*], Jules Cornetet[†], Peter Huybers[‡], Kyle C. Meng[§], and Jonathan Proctor[¶]

This file includes:

- Methods
- Data
- Figures S1-S14
- Tables S1-S3

[*]Bren School of Environmental Science and Management, UC Santa Barbara (email: tcarleton@ucsb.edu)

[†]École Normale Supérieure Paris-Saclay, Département de Sciences Sociales (email: jules.cornetet@ens-paris-saclay.fr).

[‡]Huybers: Department of Earth and Planetary Sciences, Harvard University (email: phuybers@fas.harvard.edu.

[§]Bren School of Environmental Science and Management, Dept. of Economics, and Environmental Markets Solutions Lab (emLab), UC Santa Barbara and National Bureau of Economic Research (email: kmeng@bren.ucsb.edu)

[¶]Center for the Environment and Data Science Initiative, Harvard University (email: jproctor1@fas.harvard.edu)

A Methods

A.1 SEIR model

In this section we consider a set of simulations using the standard SEIR model. The SEIR model of infectious disease has ordinary differential equations that relate susceptible (S), exposed (E), infectious (I), and recovered (R) (i.e., SEIR) compartments. We use the SEIR model to motivate and check the accuracy of our statistical approach.

We assume an SEIR model having constant rates of exposure (σ) and recovery (γ) but time-variable transmission ($\beta(t)$),

$$
\begin{aligned}
S'(t) &= -\beta(t)I(t)S(t),\\
E'(t) &= \beta(t)I(t)S(t) - \sigma E(t),\\
I'(t) &= \sigma E(t) - \gamma I(t),\\
R'(t) &= \gamma I(t).
\end{aligned}
$$

The population represented in this model is compartmentalized among susceptible (S), exposed (E), infectious (I), and recovered (R). Throughout these simulations we let $\sigma = \frac{1}{4.6}$ and $\gamma = \frac{1}{5}$ with units of $\frac{1}{day}$, following [1]. We let β take an average 0.45 $\frac{1}{people\ day}$, which corresponds to an R_0 of 2.25, consistent with previous estimates [2, 1].[1] We consider a population of 1 million individuals over 100 days. While we do not observe E, I or R directly in the COVID-19 data, we do observe the number of confirmed positive cases. We model the evolution of these confirmed cases (C) as, $C'(t) = \zeta I(t)$, allowing a portion ζ of the infectious population to be tested each time period. We let $\zeta = \frac{1}{14}$ with units of $\frac{1}{day}$ following ref. [3].[2] The dynamics of C are proportional to those of R, in that $C = \frac{\zeta}{\gamma}R$. Conceptually, this formulation allows for the possibility that a single patient tests positive multiple times, which is consistent with some reports.[3]

We define the growth rate of C as $\lambda_t^C = ln(C_t) - ln(C_{t-1})$. This growth rate is the outcome of interest in this study because it is policy relevant, observable in the COVID-19 data, and not affected by differences in testing rates between regions.[4] Although many factors may influence the time-variable nature of transmission, $\beta(t)$, here we focus on weather as the single cause of changes in transmission.

To study how shocks to β influence the growth rate of C, we first simulate the evolution of the disease in the SEIR model deterministically using the semi-implicit Euler method and analyze the impact of an idealized single-day perturbation to transmission (Fig. S12A). We let transmission vary over time with linear disturbances due to changes in weather, U: $\beta(t) = \beta_0 + \beta_1 U_t$. We parameterize U_t to generate a top-hat perturbation, equal to zero except for a single day equaling one. This leads to a proportional change in β equal to β_1 that we let take the value of 0.05 (Fig. S12B). Relative to a control run with constant β, the growth rate in I, λ^I, is seen to rapidly rise and then undergo a quasi-exponential decay (Fig. S12C,D). The response of the growth rate in C to changes in β is lagged and smoothed relative to that of I. This perturbation in ensuing growth rates from a day's change in weather is what we seek to estimate in our empirical model. The delay between the perturbation of weather and ensuing changes in growth rates

[1] Qualitative results are robust to substantial changes in σ, γ, β and ζ.

[2] Note that testing rates can be accounted for through the introduction of a parameter between 0 and 1 multiplied with ζI, as in the infection fatality literature [4]. Because we are analyzing the growth rate of C, however, such a change has no impact on the simulation results.

[3] https://www.reuters.com/article/us-health-coronavirus-who/who-says-looking-into-reports-of-some-covid-patients-testing-positive-again-idUSKCN21T0F1?il=0.

[4] We account for time-varying testing rates in our statistical model in Table S1, Section A.2.

hightlights the need to model lagged effects. The integration of this curve is the cumulative effect of a single day's change in weather on the ensuing case growth rate. In the next experiment we test the ability of temporal distributed lag regression models to capture this cumulative effect.

We use a stochastic version of the SEIR model to inform how a temporal distributed lag regression model captures the delayed effects of weather-induced shocks to transmission on the case growth rate (Fig. S13A) [46]. In this experiment, the transmission rate β is again represented as a linear function of U, but U_t is now parameterized as the sum of a sinusoid in t and Gaussian noise. This allows transmission to vary over time (Fig. S13B). Similar results are found prescribing U_t to evolve following an autoregressive moving average model with a Gaussian innovation distribution. Pooling simulated data from an ensemble of 500 runs of the stochastic SEIR model, with each 100-day run indexed by r, we estimate the effect of U on λ^I and λ^C (Fig. S13C) using a distributed lag regression model ($\lambda^C_{r,t} = c_0 + \sum_{l=\ell}^{17} \alpha_\ell U_{r,t-\ell} + \epsilon_{r,t}$). Here, c_o in an intercept and $\epsilon_{r,t}$ the error term. When estimating the regression model we use simulated data from t = 18, the earliest time point that has 17 lags of weather, to t = 45, to avoid the influence of depletion of S. The estimated lagged effects of U on λ^C and λ^I (Fig. S13D,E) represent the estimated effect of changing a single day's weather on the ensuing growth rate. The similarity in magnitude and structure of these statistically estimated effects to the dynamic response of λ^C and λ^I obtained in the idealized single-day pulse experiment using the deterministic model (Fig. S12C,D) gives confidence in our application of the lagged regression model to COVID-19 case data.

We test the ability of the distributed lag regression model to capture the cumulative effect more formally by estimating the regression model and its associated cumulative effect on 100 different simulated datasets, each containing 500 runs of the stochastic SEIR model. We calculate the cumulative effect for each regression model by summing over the 17 lagged daily coefficients, and we calculate the cumulative effect for the deterministic model by integrating the simulated perturbation in λ^C over the 17 days following the perturbation in β. The cumulative effect estimated by the distributed lag regression model closely corresponds to the cumulative effect simulated in the idealized deterministic model experiment. Specifically, the average of the estimated cumulative effect is within 8% of the value from the idealized experiment (Fig. S13F), with the differences possibly related to the deterministic versus stochastic integration performed in the two different simulations.[5] The ability of the distributed lag regression model to closely capture effects in simulated data motivates its use in observed COVID-19 case data.

In a final experiment we test the sensitivity of the estimated cumulative effect to different frequencies of changes in U. Running the stochastic SEIR simulation and temporal distributed lag regression model with weather-induced perturbations of β as in Fig. S13A, but with frequencies varying from 0.02 to 0.3 $\frac{1}{days}$ results in only small changes in the cumulative effect (Fig. S13G). This stability supports the application of our empirically determined cumulative effect, identified off of daily changes in weather and case growth rates, to simulate longer-term seasonal effects.

Though we hypothesize that the influence of weather on COVID-19 growth rates estimated in this study

[5]The influence of a perturbation of β on ensuing growth rates undergoes quasi-exponential decay. Thus, long lags are required to capture the entire cumulative effect. Using 17 lags captures 84% of the entire cumulative effect using this set of model parameters, whereas using 25 lags captures 97% of the cumulative effect. When comparing the cumulative effect estimated by the regression model to that from the idealized pulse experiment we use 17 lags in each. We note that when the estimated delay period is extended to 25 days the difference between the average cumulative effect estimated by the lag regression model and the idealized pulse experiment is reduced from 8% to 3%. The number of lags used, however, involves a trade-off between the bias and variance of the estimated cumulative effect, whereby increasing the number of lags reduces the bias but increases the variance of the estimated effect. The specification of a lag length of 17 days for the empirical model is based on existing empirical estimates of the delay interval between exposure and case confirmation (Section A.2) and is supported by our model simulations to capture the preponderance of the effect. We show robustness of the cumulative effect to changes in lag length in Fig. S7.

are due to changes in transmission, we do not estimate the effect of weather on transmission directly because doing so would require additional assumptions. Changes in transmission have a complex relationship with changes in the growth rate. Even in the simple case of the model being in equilibrium in the disease-free limit (i.e. approximately all of the population being susceptible) the populations of E, I, and C grow at an asymptotic rate equal to $\lambda = \frac{-(\sigma+\gamma)+\sqrt{(\sigma-\gamma)^2+4\sigma\beta}}{2}$ [6]. Solving for β, differentiating and re-arranging gives: $\frac{\partial\lambda}{\partial\beta} = \frac{1}{\eta}$ where $\eta = (1 + \frac{\gamma}{\sigma} + \frac{2\lambda}{\sigma}) > 1$. This shows that an equilibrium change in β causes a damped equilibrium change in λ, and that the degree of damping is a function of the model parameters.

The dependence of $\frac{\partial\lambda}{\partial\beta}$ on other model parameters, which are imprecisely known, complicates estimation of the impact of weather on transmission even in this relatively simple equilibrium setting. Stochastic changes in β over time due to changes in weather introduce further complexity to the relationship between transmission and the case growth rate. Thus, we estimate the impact of weather directly on the growth rate and leave estimation of weather effects on transmission to future work. We are unaware of an analytical solution for the growth rate of I, or C, under a time-variable β, and note that obtaining such a solution would be useful for purposes of optimizing inferences from changes in the growth rate.

Note that the shapes of the lagged responses seen in both the stochastic and deterministic models are determined by the assumptions of the SEIR model (e.g. an exponentially distributed infectious period). Given that the dynamics of COVID-19 is unlikely to satisfy these assumptions, we should not expect lagged responses recovered from the data to precisely match simulated responses. Further, while the primary mechanism through which weather is thought to impact COVID-19 growth is through changes in transmission, which motivates these simulations, it is possible that weather also impacts the testing rate, recovery rate, or incubation period. Thus, the estimated impacts of weather on λ^C should be interpreted as the combined effect of potentially multiple channels – both biological and social.

A.2 Statistical model

The SEIR simulations in Section A.1 suggest that a distributed lag regression model can capture delayed effects of weather-induced shocks to transmission on the COVID-19 growth rate. Implementing such a statistical model on actual data, however, requires additional consideration of the various potential confounding factors that can influence the true data generating process, but which were not included in the simple SEIR model we examine.

In general, there are four challenges to causal estimation in this setting. First, surface weather conditions vary systematically as one moves away from the equator towards higher latitude locations. For example, temperatures and specific humidity both decline at higher latitudes. Because similar latitude-dependent gradients exist for other potentially relevant environmental conditions like natural disaster exposure and socio-economic indicators like GDP, a cross-sectional analysis of local mean climate conditions and COVID-19 infection rates may be biased by such confounding factors. Second, for a given location, environmental conditions generally trend over the course of a calendar year. Because COVID-19 infection rates trend as well, such temporal dependence may also confound empirically estimated weather effects on COVID-19 with other gradually evolving determinants of infection. Third, many local environmental conditions are strongly correlated. These correlations would confound causal estimates if key variables are omitted from the analysis. Lastly, any convincing causal estimate must take into account the time delay between COVID-19 transmission and detection.

This study takes a quasi-experimental statistical approach that addresses such potentially confounding factors in order to isolate random variation across a set of environmental conditions: UV, temperature,

humidity, and precipitation. This "reduced-form" empirical approach is agnostic regarding the mechanisms through which climate variables govern the growth rate of cases, but by providing plausibly causal estimates of the role that each plays in the evolution of the virus, allows one to make counterfactual simulations of future conditions under alternative environmental conditions. Furthermore, such estimates provide empirical grounding for the parameters of more process-based models like the SEIR model.

Specifically, we estimate a longitudinal (i.e. panel) regression model using daily confirmed COVID-19 cases from 173 countries from January 01, 2020 to April 10, 2020. Our outcome of interest is the growth rate of cumulative COVID-19 cases in administrative (i.e., national/subnational) unit i between days t and $t-1$, $\lambda_{it}^C = \ln C_{it} - \ln C_{it-1}$. Because of the delay between initial COVID-19 exposure and confirmed detection (Section A.1), we model the growth rate in cumulative COVID-19 cases using the following distributed lag model:

$$\lambda_{it}^C = \sum_{\ell=0}^{\ell=L} \alpha_\ell^{UV} UV_{i,t-\ell} + \sum_{\ell=0}^{\ell=L} \alpha_\ell^{T} T_{i,t-\ell} + \sum_{\ell=0}^{\ell=L} \alpha_\ell^{H} H_{i,t-\ell} + \sum_{\ell=0}^{\ell=L} \alpha_\ell^{P} P_{i,t-\ell} + \theta' \mathbb{Z}_{it} + \epsilon_{it}, \qquad \text{(S1)}$$

where for administrative unit i and day t, $UV_{i,t-\ell}$, $T_{i,t-\ell}$, $H_{i,t-\ell}$, and $P_{i,t-\ell}$ are population-weighted daily average UV (in kJ/m^{2}hour), temperature (in degrees centigrade), specific humidity (in %), and precipitation (in mm), respectively, observed ℓ days ago. In robustness checks (Fig. S6), we show that specific and relative humidity generate very similar results. Joint estimation of these local environmental variables addresses concerns regarding their correlation structure. The estimation of multiple lagged effects allows for a data-driven way of capturing the delay between COVID-19 transmission and eventual detection. Because daily weather variables observed in a given location are highly serially-correlated, it is often difficult to discern a statistically precise time pattern of lagged effects when including lagged daily UV, temperature, humidity, and precipitation. To reduce this noise, we construct lagged weather terms that are averaged over 3-day periods, such that the lag index indicates 3-day averages, $\ell \in \{$0-2, 3-5, 6-8, 9-11, 12-14, 15-17$\}$. We stop at 17 days because it covers the range of 6-14 day delays between transmission and COVID-19 case confirmation detected in the existing literature [35, 36, 37, 38]. One can always include additional lag terms, but there is a potential bias-variance trade-off between capturing a sufficiently long interval over which most cases should be recorded and introducing additional lagged variables: with fixed data, more lagged terms result in a noisier cumulative effect. Moreover, if the true effect for those additional lagged terms is zero, their inclusion in the model would contribute statistical noise without added signal. In a robustness check, we also include leads of 3-day average values for UV, temperature, humidity and precipitation and addition lags up to 20 days later.

We are interested in quantifying the total effect of environmental exposure in a single period as it manifests over subsequent time periods. In a temporal-distributed lag model like Eq. S1, this total effect is captured by the sum of lagged effects for each weather variable, or the "cumulative effect." To see this, observe that the effect of, say, UV_{it} on subsequent COVID-19 growth rates is:

$$\frac{\partial \lambda_{it}^C}{\partial U_{it}} = \alpha_0^{UV}, \frac{\partial \lambda_{it+1}^C}{\partial U_{it}} = \alpha_1^{UV}, \ldots, \frac{\partial \lambda_{it+L}^C}{\partial U_{it}} = \alpha_L^{UV},$$

such that the total (or cumulative) effect of period UV_{it} on subsequent COVID-19 growth rates up to 17 days later is $\sum_{\ell=0}^{L} \alpha_\ell^{UV}$. The estimated uncertainty in the cumulative effect takes into account the variances

of each lagged effect as well as their covariances, specifically:

$$var(\sum_{\ell=0}^{L} \alpha_\ell^{UV}) = \sum_{\ell=0}^{L} var(\alpha_\ell^{UV}) + \sum_{\ell \neq m} cov(\alpha_\ell^{UV}, \alpha_m^{UV}).$$

Importantly, in calculating the cumulative effect, we include all estimated lagged effects within the 17 day interval, including effects that are imprecisely estimated. This is because with heterogeneity in delay intervals across individuals, one would expect population-level studies such as ours to detect population-weighted lagged effects throughout the 17 day interval. As such, our approach must include such noisy estimates as they reflect existing uncertainties. We calculate the cumulative effect and its standard error separately for each weather variable using the estimated coefficients and covariance matrix from the model in Eq. S1.

To isolate plausibly random variation in weather conditions [11, 21], we include $\mathbb{Z}_{it}$, a vector of semi-parametric controls. In our baseline specification, $\mathbb{Z}_{it}$ includes a full set of national/subnational unit-specific dummies, which remove any time-invariant differences in growth rates of COVID-19 cases and environmental variables across administrative units. These spatial "fixed effects" address the concern that baseline population characteristics (e.g. economic activity, population density) may be correlated both with COVID-19 infection rates and with average weather conditions. Second, $\mathbb{Z}_{it}$ includes day-specific dummies to remove any common global determinants of COVID-19 growth rates. These temporal fixed effects account for global daily circumstances that may influence COVID-19 growth rates such as WHO's declaration of COVID-19 as a global pandemic. To account for local trends in both COVID-19 growth rates and weather during this period, $\mathbb{Z}_{it}$ includes country-by-week dummies, which flexibly account for country-specific temporal trends and shocks in COVID-19 growth rates and weather. Importantly, these dummy variables capture gradually occurring local trends across the globe as COVID-19 evolves. The influence of adding this suite of controls on the residual variation in UV, temperature, and COVID-19 growth rates is shown visually for two selected regions in Fig. 2A. Finally, we cluster standard errors, ϵ_{it} at the administrative level. This allows for data-driven heteroskedasticity and serial correlation of arbitrary form in the error terms of each administrative unit.

The results of several robustness checks are given in Table S1 wherein a suite of controls are alternatively examined. Examined controls include the number of days since the initial outbreak of COVID-19 in each location (col. 1), country-specific linear trends (col. 2), country-by-week fixed effects (col. 3, and in combination with others in cols. 4-6), a placebo "lead" weather variable measuring future exposure (col. 4), timing of policies such as school closures (col. 5), stringency of the COVID-19 testing regime at national level (col. 6), increased spatial resolution of our week-specific dummies to include subnational administrative unit-by-week fixed effects (col. 7), and use of country-by-day fixed effects, as opposed to country-by-week, (col. 8), which requires dropping all data from countries without subnational COVID-19 records, as daily weather variables are collinear with these dummy variables.

To control for social distancing policies (Table S1, col. 5), which vary across space and time, we add to the regression model in Eq. S1 a dummy variable equal to 1 when any one of three policies are in place: school closures, work from home ordinances, and event cancellations (Section B.3). To control for changes over space and time in the degree of COVID-19 testing (Table S1, col. 6), we use national-level records from OxGCRT (Section B.3) that categorize each country's testing regime. Categories are: No testing policy (coded as 0), only testing those who have symptoms *and* meet specific criteria, such as being essential workers or coming into contact with a known case (coded as 1); testing of anyone showing COVID-19 symptoms (coded as 2); and open public testing, such as drive-through testing, available to asymptomatic people (coded as 3).

A variable indicating which regime a country falls into on any given day is added to the regression model shown in Eq. S1. Modelling this ordinal variable as four binary variables gives nearly identical results.

Finally, we estimate a Poisson Pseudo-Maximum Likelihood estimator, in place of the ordinary least squares regression shown in Eq. S1 (col. 9, and col. 5 of Fig. S5). We do so for two reasons: first, the distribution of new cases is very skewed; second, if climate conditions operate solely through the transmission parameter β (Section A.1), changes in climatic conditions cannot lead to negative growth rate effects, as transmission cannot be negative. Because the former concern is not empirically large (in Fig. S14, we show that our residuals from estimation of Eq. S1 are approximately normally distributed), and because climate variables may influence growth rates through transmission as well as other channels, such as behavior regarding testing, we include this model as a robustness check only. The estimating equation relates new cases realized between day $t-1$ and day t, denoted ΔC_{it}, to lagged climatic exposure as follows:

$$\Delta C_{it} = exp\left(\sum_{\ell=0}^{\ell=L} \alpha_\ell^{UV} UV_{i,t-\ell} + \sum_{\ell=0}^{\ell=L} \alpha_\ell^{T} T_{i,t-\ell} + \sum_{\ell=0}^{\ell=L} \alpha_\ell^{H} H_{i,t-\ell} + \sum_{\ell=0}^{\ell=L} \alpha_\ell^{P} P_{i,t-\ell} + \theta' \mathbb{Z}_{it} + \rho C_{it-1}\right) + \epsilon_{it}. \quad \text{(S2)}$$

We control for lagged cumulative cases C_{it-1} as new cases ΔC_{it} are proportional to the level of infected people in the population (Section A.1). All other variables are defined as in Eq. S1. While standard Poisson models impose that the first and second moments of the outcome be equal, we address this overdispersion issue by clustering standard errors at the administrative unit level. This adjustment relaxes the assumption of equal first and second moments by allowing arbitrary forms of within-administrative unit heteroskedasticity and serial correlation in the error term ϵ_{it} [13].

Eq. S1 implicitly assumes a linear relationship between environmental conditions and COVID-19 growth rates. To explore potential nonlinearities in these relationships without requiring polynomial terms for every lagged weather variable in Eq. S1, we estimate an alternative model in which we impose that weather conditions have constant linear (Eq. S3) and quadratic (Eq. S4) effects throughout the 17-day delay period:

$$\lambda_{it}^{C} = \alpha_1^{UV} \sum_{\ell=0}^{\ell=L} UV_{i,t-\ell} + \alpha_1^{T} \sum_{\ell=0}^{\ell=L} T_{i,t-\ell} + \alpha_1^{H} \sum_{\ell=0}^{\ell=L} H_{i,t-\ell} + \alpha_1^{P} \sum_{\ell=0}^{\ell=L} P_{i,t-\ell} + \theta' \mathbb{Z}_{it} + \epsilon_{it}, \quad \text{(S3)}$$

$$\begin{aligned}\lambda_{it}^{C} =& \alpha_1^{UV} \sum_{\ell=0}^{\ell=L} UV_{i,t-\ell} + \alpha_2^{UV} \sum_{\ell=0}^{\ell=L} UV_{i,t-\ell}^2 + \alpha_1^{T} \sum_{\ell=0}^{\ell=L} T_{i,t-\ell} + \alpha_2^{T} \sum_{\ell=0}^{\ell=L} T_{i,t-\ell}^2 \\ &+ \alpha_1^{H} \sum_{\ell=0}^{\ell=L} H_{i,t-\ell} + \alpha_2^{H} \sum_{\ell=0}^{\ell=L} H_{i,t-\ell}^2 + \alpha_1^{P} \sum_{\ell=0}^{\ell=L} P_{i,t-\ell} + \alpha_2^{P} \sum_{\ell=0}^{\ell=L} P_{i,t-\ell}^2 + \theta' \mathbb{Z}_{it} + \epsilon_{it}. \quad \text{(S4)}\end{aligned}$$

For each weather variable, Fig. S10 plots three relationships between that weather variable and the cumulative change in the COVID-19 growth rate over the 17 day delay period. The solid line and 95% confidence interval shows the cumulative effect from our distributed lag model in Eq. S1. The dashed line shows the analogous cumulative effect using the linear constant effects model shown in Eq. S3. The linear relationship is nearly identical to that obtained from our distributed lag model. The dotted-dashed line shows the quadratic relationship from the quadratic version of the constant lagged effects model shown in Eq. S4. There does not appear to be strong nonlinearities in the UV, temperature, or specific humidity effects. Precipitation effects appear to exhibit nonlinearities but this relationship is not statistically significant.

In Fig. 3A,C, we show cumulative effects of lagged responses of COVID-19 to UV, temperature, and

specific humidity. We additionally show heterogeneity in this cumulative effect across policy regimes (purple diamonds) and duration of outbreak (green squares). The former coefficients are generated by estimating a version of Eq. S1 in which each weather variable lag is interacted with the corresponding lagged value of a policy dummy variable. This dummy variable is equal to 1 when any one of three policies are in place: school closures, work from home ordinances, or event cancellations (Section B.3). "Pre-policy" cumulative effects are then computed using estimated lagged effects of each weather variable when the policy dummy is set to 0; in contrast, "post-policy" cumulative effects are computed using estimated coefficients when the policy dummy is set to 1. Similarly, to recover heterogeneity by duration of outbreak, we define a dummy variable equal to 1 when an observation for a given location and day occurs at least 30 days after the first recorded COVID-19 case within that population. This dummy variable is then interacted with each lagged weather variable in a regression otherwise identical to Eq. S1. Cumulative effects for the first month of outbreak are computed using estimates of lagged weather variable effects when the outbreak duration dummy is 0; "after first month" cumulative effects are similarly computing by setting the outbreak duration dummy to 1.

A.3 Smooth fits to estimated lags

In Eq. S1, we non-parametrically estimate a set of lagged coefficients for each weather variable, allowing for arbitrary dynamic structure in the effect of climatological conditions on subsequent COVID-19 growth rates. While this approach is highly flexible, it is demanding on the data, leading to noisy estimates of the lag structure. Because the true lagged response of the COVID-19 growth rate to environmental factors is likely to be smooth over time (ection A.1), in Fig. 3B we show a smoothed fit to these estimated lag coefficients. To do so, we fit a restricted cubic spline with four degrees of freedom to lag coefficients estimated in Eq. S1 and shown for the central estimate in col. 1 of Fig. S5. Observations are weighted by their empirical precision. Note that when comparing the magnitudes of the fitted responses and the lagged coefficients, that each lag coefficient shows the effect of changing average environmental conditions over three days, and are thus three times larger than the the fitted response, which shows the effect at each lag of changing environmental conditions for a single day.

A.4 Seasonal simulations

To conduct seasonal simulations, we calculate the daily seasonal climatology of UV, temperature, and specific humidity by averaging daily data from the ERA5 reanalysis product over the years 2015 to 2019 (Section B.4). In Fig. 4B-C, we represent the monthly effect of each climate variable on the predicted COVID-19 growth rate as the product of the cumulative effect of each variable estimated in Eq. S1 and the average hourly weather over each calendar month. To capture differential seasonality across time, we show in Fig. 4B-C the difference between predicted growth rates under the climatology of January and under the climatology of June. In Fig. S11, we show the analogous difference between June and December.

To compare the estimated influence of seasonality on COVID-19 growth rates to that of social distancing policies, we use estimates from ref. [16]. In this paper, the authors estimate the impact of a collection of social distancing policies on daily growth rates in confirmed COVID-19 cases (i.e., the authors use the same outcome variable as used throughout this analysis) across six countries: Iran, China, South Korea, United States, Italy, and France. We use the authors' estimates of the impact of all social distancing policies combined (Fig. 2b in ref. [16]); omitting the impact of policies in China during the first week of lockdown (which is statistically insignificant), effect sizes range from -0.2 to -0.49 across countries. These magnitudes

imply that the imposition of social distancing policies lowers daily COVID-19 growth rates by 20 to 49 percentage points. In comparison, the largest influence of seasonality that we recover for each region of the world is: 7.75 percentage points in the extra-tropical Northern Hemisphere, 7.72 percentage points in the extra-tropical Southern Hemisphere, and 0.57 percentage points in the tropics.

B Data

B.1 COVID-19 case data

To statistically estimate a plausibly causal, global relationship between local weather variations and the pattern of COVID-19 transmission we construct a harmonized global dataset of geolocated daily confirmed COVID-19 cases. We use data obtained from national governments, subnational authorities and newspapers, ultimately covering 3,235 administrative units across 173 countries and five continents.

B.1.1 National-level COVID-19 data

For all countries for which subnational records were not publicly available at the time of writing, we use national data assembled by the Johns Hopkins University Center for Systems Science and Engineering [60] for the period between January 01, 2020 to April 10, 2020.[6] We omit observations from the Diamond Princess cruise ship, due to uncertain weather exposure of the passengers.

B.1.2 Subnational COVID-19 data

Table S2 describes the characteristics of and sources for the COVID-19 case data we collected and compiled at the subnational level. Below, we provide some additional detail regarding data cleaning and manipulation for each individual country.

In most countries, we directly obtain subnational reports of the daily number of newly confirmed COVID-19 cases. To compute cumulative case counts at the daily level, we then compute cumulative sums for each subnational unit. When only cumulative COVID-19 cases are available on a daily basis, we take first differences in the time series for each subnational unit to obtain the number of new cases detected on each day. If not mentioned otherwise, we assume that missing values after the start of the epidemic in a given subnational unit correspond to zero new cases. Because we obtain subnational case data from ref. [16] for Iran and China, we follow their imputation method for addressing missing data in these two countries; details of this method are described by the authors.[7]

In many countries, additional data cleaning was required to accurately and consistently match new cases to the day on which they were detected, as opposed to the day on which they were reported. Harmonizing the data in this way reduces measurement error when estimating a common lagged response across the pooled sample. To do so, we track the dates and hours of the day on which new cases were released; when new cases are obtained from morning reports (before noon), we assign cases to the previous calendar day. Details on such corrections are presented below for each country. We compare our compiled subnational COVID-19 case data with case data reported at national level by John Hopkins University (JHU) and by the European

[6] Available at https://github.com/CSSEGISandData/COVID-19 and accessed via https://github.com/RamiKrispin/coronavirus.

[7] Available here: https://www.dropbox.com/s/1xvskw6dark53l0/2020321_GPL_COVID_appendix.pdf?dl=0.

Center for Disease Prevention and Control (ECDC) (Fig. S1).[8]

Austria (1st administrative level) See Table S2 for details. Because no alternative archived reports are available for Austria, we verify our data against data stored in the GitHub public repository "covid-19-eu-data," which provides time series for COVID-19 cases in European countries based on the scraping of official reports.[9] Our figures correspond to the official afternoon reports.

Belgium (1st administrative level) See Table S2 for details. We append data from two versions of the Wikipedia article "2020 coronavirus pandemic in Belgium." The current Wikipedia page (as of April 13, 2020) provides data starting on March 1, 2020. Data from January 30 to March 1, 2020 were webscraped from an earlier version of the same article (accessed on April 6, 2020). The distribution of cumulative cases on March 1, 2020 in the current article matches those from the previously collected time series. We drop data for April 7 and April 8, 2020, as we detect a discontinuous drop in new cases and increase in missing values. Our numbers have been verified against Sciensano data[10] for the days covered by both sources.

Brazil (1st administrative level) See Table S2 for details. For São Paulo, we add an additional case to the cumulative case count for February 25, 2020, based on newspaper reporting that a single case was already present.[11] We confirm that our data match the official source.

Chile (1st administrative level) See Table S2 for details. As stated in the daily reports, the information provided in official publications document cases reported on the previous day. In order to associate new cases with the date of detection and not the date of announcement, we correct the data webscraped from Wikipedia by lagging each date by one.

France (1st administrative level) See Table S2 for details. France overseas territories have been removed from the analysis due to the low number of cases at the time of data collection (116 cases distributed over 7 territories on March 25, 2020). On March 25, 2020, the French Public Health Agencies stopped publishing COVID-19 cases data at the regional level. Because the cumulative number of cases proposed by Wikipedia after this date is systematically below official figures at the national level and because the corresponding sources are not verifiable, we retain data only until March 25, 2020. It was not possible to find archived reports for French COVID-19 cases at the regional level for data verification purposes. We thus compare our data against the time series offered on the open platform for French public data.[12] The number of cases reported in both datasets are very similar.

Germany (1st administrative level) See Table S2 for details. While the Robert Koch Institute (RKI) publishes case data for COVID-19, these data do not exist prior to March 3, 2020. We therefore rely on webscraped data from Wikipedia, which is obtained from newspaper articles, and validate these data against those available from RKI. Between March 4 and March 10, 2020, the Wikipedia figures do not match the

[8] ECDC data have been directly downloaded from the ECDC website: https://www.ecdc.europa.eu/en/publications-data/download-todays-data-geographic-distribution-covid-19-cases-worldwide.

[9] Available at: https://github.com/covid19-eu-zh/covid19-eu-data/.

[10] Available at: https://epistat.wiv-isp.be/covid/.

[11] See for instance: https://newslab.com.br/primeiro-caso-do-covid-19-e-confirmado-no-brasil/ (in Portuguese).

[12] Available at: https://www.data.gouv.fr/en/datasets/fr-sars-cov-2/.

Robert Koch Institute (RKI) reports. Hence, we recode manually the series for these days, using the official data from RKI. Due to the relative novelty of the epidemic at that time, some reports are inconsistent with the figures presented in preceding reports. In case of such inconsistencies, we consider the most recent report as the most reliable one and correct our number of cases accordingly.

On March 17, 2020, the RKI stopped updating its data manually and switched to an automated process based on the data electronically transmitted up to 11:00pm on the previous day.[13] After this date, we correct the date in our data by lagging records by one day, in order to retrieve the accurate day of detection. For March 17, we sum new cases recovered from the reports of March 17 (confirmed cumulative infections up to March 17, 2020, 11:00pm) and March 18 (confirmed cumulative infections up to March 18, 2020, 0:01am).

Both Wikipedia and the Robert Koch Institute point out that some reports are missing, but do not consider this information in their computation of the cumulative cases series. The number of total confirmed cases reported is thus artificially stable for the dates in which reports are known to be missing. We therefore code as missing the new cases and confirmed cumulative cases for North Rhine-Westphalia on March 10 and 11, for Saxony-Anhalt on March 26 and 28 and for Baden-Wurttemberg and Hesse on March 27, to account for the absence of data collection for these dates.

Our panel data on cumulative cases begins with 14 cases in Bavaria on February 24, 2020. As it is unlikely that these 14 cases appeared all at once, we set the initial value of our new cases series as missing.

Iran (1st administrative level) See Table S2 for details. The number of new cases for all regions on March 2 and March 3, 2020, are missing, due to an absence of reporting. These missing values have been imputed following the method implemented by ref. [16], who used and verified the same source of data.

Netherlands (1st administrative level) See Table S2 for details. The table we obtain from Wikipedia associates the number of new confirmed cases with the day on which they were first announced. As official reports for the Netherlands are published in the morning, we correct the date by lagging reported cases by one day, relative to that provided by Wikipedia.

Portugal (1st administrative level) See Table S2 for details. Official reports until March 9, 2020, were published in the late afternoon. On March 10, however, the Directorate-General of Health began to publish morning reports compiling the number of total confirmed cases up to midnight on the previous day. As the Wikipedia article we webscrape does not consider this change, we correct the date for each day after March 10, 2020, by lagging the case records by one day. For March 10 itself, we discard the row associated to March 10 on Wikipedia (2 new confirmed cases) and only keep the one associated to the report published on March 11.

These data have been verified against official reports. For March 2, 2020, as there was no official report at this stage of the epidemic, we verified the information in newspapers documenting the first occurrence of the epidemic in Portugal.[14]

South Korea (1st administrative level) See Table S2 for details. The time at which official counts have been released changes over the sample period. Until March 1, 2020, updates to case records often occur

[13] https://www.rki.de/DE/Content/InfAZ/N/Neuartiges_Coronavirus/Situationsberichte/2020-03-17-en.pdf?__blob=publicationFile.

[14] See for instance: https://www.reuters.com/article/us-health-coronavirus-portugal/portugal-registers-first-two-cases-of-coronavirus-sic-television-idUSKBN20P1BB.

twice per day. The confirmed new cases announced in each report are those that have been detected since the last report: the new cases announced in the afternoon have thus been detected within the day, since the morning count. From January 30 to March 1, we group the morning count with the afternoon count of the previous day, to get a detection period covering 9:00am on the previous day to 9:00am on the current day. On these dates, the date of the afternoon count has been kept in order to match the day of detection. On March 1, we sum the cases obtained from the afternoon report to the new cases extracted from the evening report, released at midnight. From March 2 forward, the Korean Center for Disease Control (KCDC) publishes morning reports containing information for the previous day, from 00:01am to 11:59pm. As the Wikipedia article correctly considers this change, no change has been made on the date of new cases for March 2 onward.

Spain (1st administrative level) See Table S2 for details. Spanish reports are published in the morning and contain information about the previous day. We thus correct the date in the data obtained in a public GitHub repository by lagging case counts by one day in order to accurately recover the day of detection. The number of new cases for Ceuta and Melilla have been summed to match the spatial shapefile we use for aggregating gridded climate data.

Sweden (1st administrative level) See Table S2 for details. Swedish official data regarding the COVID-19 epidemic are updated daily at 11:30am. To match the day of detection, we lag the case count reported by Wikipedia by one day. The first COVID-19 case in Sweden was observed on February 3, 2020, in the Jönköpping region. As no additional cases were detected during the three weeks following the occurrence of this first case, we drop it from our continuous confirmed new cases series, which begin on February 25, 2020. However, we keep it when computing the number of cumulative cases.

United Kingdom (1st administrative level) See Table S2 for details. Because there were about 1700 cases that had not been precisely located within England at the time of initial data collection (April 6, 2020), we aggregated case data to the level of England, Wales, Scotland, and Northern Ireland, instead of using National Health System (NHS) regions. In the United Kingdom, confirmed new and cumulative COVID-19 cases are announced in the morning. We thus lag cases reported by Wikipedia by one day to accurately reflect date of detection. Since the date of our initial data collection, Public Health England has published complete time series at the NHS region level and at the county level. We checked our data against these series and verified that they were nearly identical.

China (2nd administrative level) See Table S2 for details. We drop 29 cities that could not be merged with climate data, based on publicly available geographic shape files. It was not possible to check the time at which city reports were issued. As a result, we consider the date of announcement as the date of detection. Missing data have been imputed following the interpolation method performed by ref. [16].

Italy (2nd administrative level) See Table S2 for details. The number of cases is updated daily at the end of the afternoon. Our data are almost identical to those obtained at national level from JHU, although JHU data display a break on March 12 due a to a delay in JHU updates, an issue reported for several countries in this dataset.[15]

[15] Up to April 17, 2020, there are more than 10 open issues on this topic on the CSSEGISandData/COVID-19 public repository managed by JHU. See for instance: https://github.com/CSSEGISandData/COVID-19/issues/619.

United States (2nd administrative level) See Table S2 for details. For the United States, all cases are counted on the date they are first announced, and cases are located at the place where they are treated. Although the *New York Times* mostly uses the official counties as the unit of analysis, a few exceptions are worth mentioning:[16]

- The five boroughs of New York City have been gathered under the label "New York City";
- The COVID-19 cases for Cass (MO), Clay (MO), Jackson (MO) and Platte (MO) counties are exclusive of the cases detected in Kansas City, shown on their own. We drop observations under the label "Kansas City, Missouri," which does not correspond to any official county;
- All cases for Chicago are reported within Cook County (IL).

We download all the data from the *New York Times* repository, and keep only the cumulative cases, computing new cases using first differences. Because some county-level series start with strictly positive numbers (up to 37 cases on the first day), we define the first observation of each new cases series as missing, but keep this number in our cumulative cases series.

B.2 Population data

Our main outcome variable is the first difference in the natural logarithm of daily cumulative cases per 1 million people. At the national level, we use the country-level population in 2018 (the most recent year available) from the World Bank's World Development Indicators.[17] No homogeneous source of data have been found at the subnational level. We therefore obtain the most recent data available from each country's national office of statistics. Detailed information on each source can be found in Table S3.

B.3 Policy and COVID-19 testing data

We collect data on the intensity of COVID-19 testing and on social distancing policies from ref. [16] and the Oxford COVID-19 Government Response Tracker (OxCGRT) [15], described below. Briefly, from these data we obtain – for each administrative unit in our analysis – a policy variable, which is equal to 1 if any policy that closes schools, closes workplaces, or cancels public events is implemented, and 0 otherwise. We also obtain an ordinal variable with four levels that describes the intensity of testing.

Social distancing policy data from ref. [16] The first set of variables we use has been compiled by ref. [16] in their study on the effect of large-scale anti-contagion policies on the COVID-19 pandemic. The authors collect policy data at the subnational scale for China (2nd administrative unit), France (1st administrative unit), Iran (1st administrative unit), Italy (2nd administrative unit) and the United States (1st administrative unit). We directly merge these policy data into our database for the corresponding dates. For the United States, we match each county within a state to the state-level (i.e. 1st administrative level) data from ref. [16], as no county-level policy data are available.

We use three variables from this study, which match with variables within the OxCGRT dataset. They are defined in in ref. [16] as:

[16] Detailed information are provided on the COVID-19 GitHub repository of the *New York Times*.
[17] Available at: http://data.worldbank.org/data-catalog/world-development-indicators.

1. **school_closure**: "A policy that closes school and other educational services in that area."

2. **work_from_home**: "A policy that requires people to work remotely. This policy may also include encouraging workers to take holiday/paid time off."

3. **event_cancel**: "A policy that cancels a specific pre-scheduled large event (e.g. parade, sporting event, etc.). This is different from prohibiting all events over a certain size."

All these variables are binary variables. They take the value 1 starting on the day a policy is implemented, and 0 if the policy is not implemented. Only policies that are legally enforced are considered here; optional policies and non-binding recommendations from governments are not included.

The Oxford COVID-19 Government Response Tracker (OxCGRT) The second set of policy variables we use contains data at the national level for over one hundred countries across the world. The original dataset has been compiled by a group of researchers affiliated to the Blavatnik School of Government at Oxford [15].[18]

To match the data from ref. [16] we collect three variables from this database:

1. **School_closing**: "Record closings of schools and universities".

2. **Workplace_closing**: "Record closings of workplaces".

3. **Cancel_public_events**: "Record cancelling public events".

These variables were initially coded as categorical variables taking the values: not implemented, optional and legally enforced.[19] We recode these variables as binary variables to match the format of policy data from ref. [16], setting the value to 1 if the policy is legally enforced and zero otherwise.

COVID-19 testing data OxGCRT provides a measure of testing access and the testing efforts deployed by national governments. This ordinal variable takes on the following values: absence of testing policy (0); very restricted access to testing, conditional on both symptoms and past and present exposure or professional situation (1); testing only individuals with symptoms (2); and deployment of open public testing (3).

Final data manipulation Both the the data from ref. [16] and the OxCGRT data have been merged with our epidemiological dataset using the corresponding dates and administrative levels, giving preference to subnational data when available. From these data we create a binary policy variable that takes the value of 1 if any policy closing schools, workplaces or public events was enacted and 0 otherwise.

B.4 Weather data

We use the ERA5 reanalysis product from European Centre for Medium-Range Weather Forecasts (ECMWF), which provides daily gridded weather variables at the 0.25° latitude by 0.25° longitude resolution [17].[20] Specifically, for January 01, 2020 to April 10, 2020, we collect hourly downward UV radiation at the surface

[18] Data can be downloaded here: https://www.bsg.ox.ac.uk/research/research-projects/coronavirus-government-response-tracker The working paper presenting the construction of the database can be found at https://www.bsg.ox.ac.uk/sites/default/files/2020-04/BSG-WP-2020-031-v4.0_0.pdf.

[19] The original codebook associated with this database is available at https://www.bsg.ox.ac.uk/sites/default/files/2020-04/BSG-WP-2020-031-v4.0_0.pdf.

[20] Available at: https://cds.climate.copernicus.eu/cdsapp#!/dataset/reanalysis-era5-single-levels?tab=overview.

(in J/m^{2}hour), 2-meter temperature (in degrees centigrade), total 2-meter precipitation (in mm), and 1000 hPA specific humidity (in kg/kg). 2-meter and 1000 hPA roughly correspond to conditions near the earth's surface. In robustness checks (Fig. S6), we include relative humidity, which we sample at 1000 hPA. We average UV, temperature, and specific humidity across hours in the day to obtain daily average measures, while we sum precipitation across hours in the day to obtain daily total precipitation.

We link gridded weather data to administrative-level COVID-19 cases by aggregating grid cell information over administrative (e.g. country, province, or county) boundaries. To capture climatic conditions reflective of population exposure, we average across grid cells weighting by the cross-sectional gridded distribution of population in 2011 from LandScan [18]. For example, administrative-level daily population-weighted average temperature is computed as $T_{it} = \sum_{g \in i} \omega_{gi} T_{gt}$, where g indicates grid cell, i indicates an administrative unit, and ω_{gi} is the share of administrative unit i's population that falls within grid cell g.

To estimate projected seasonal conditions and their influence on COVID-19 transmission, we construct daily gridded UV radiation, 2-meter temperature, and 1000 hPA specific humidity from ERA5, as described above, over the last five years (2015-2019). Using the average conditions for each calendar day over the past five years as a proxy for expected seasonal variation through 2020 and into early 2021 (Fig. S4), we compute daily averages across all five years of daily temperatures at both grid cell level (Fig. 4C) and aggregated to latitudinal groups (Fig. 4A), in the latter case using the same aggregation method described above.

C Supporting Figures

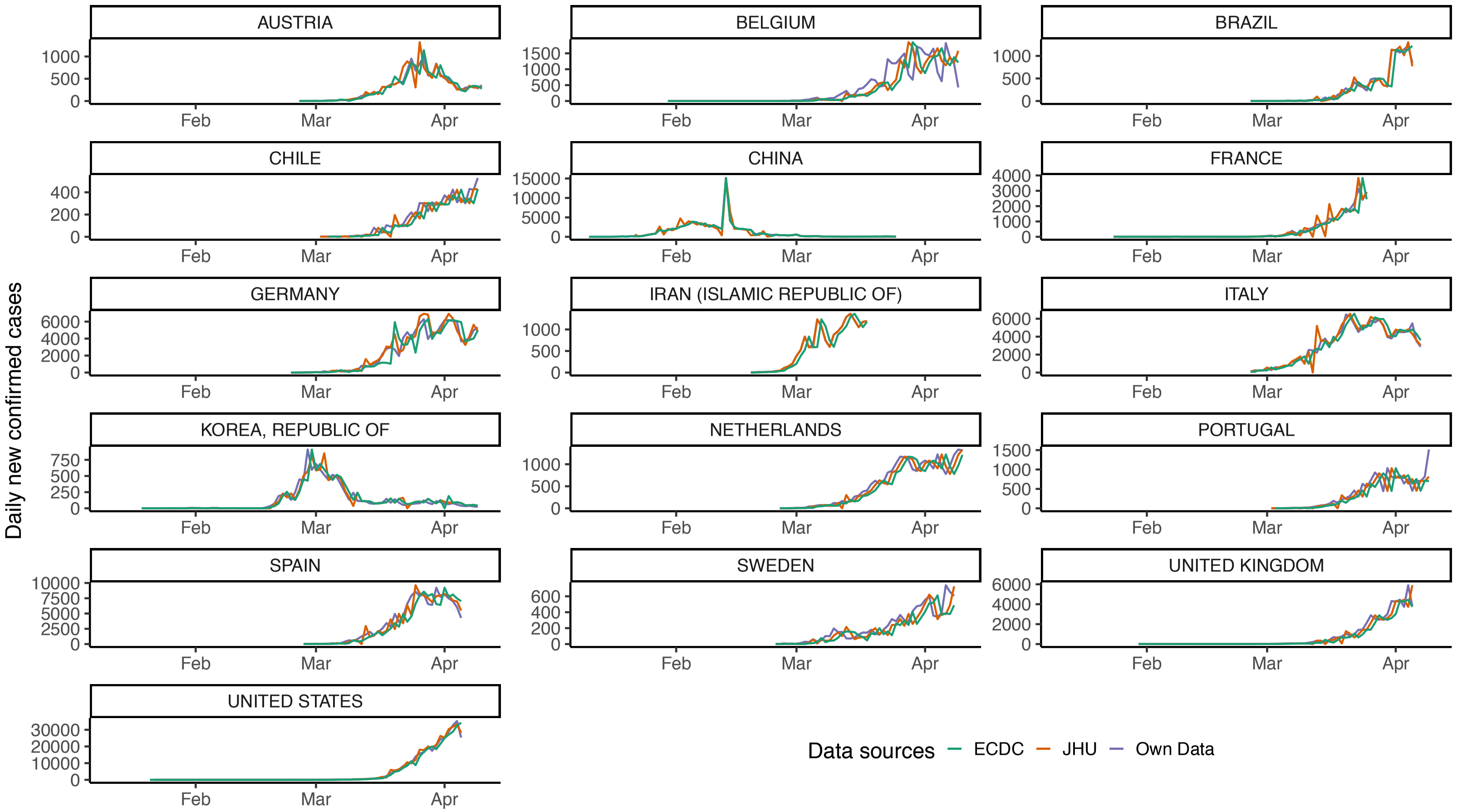

Figure S1: Comparison of assembled subnational data to JHU-CSSE and ECDC national-level data sources. To verify the quality of our assembled subnational COVID-19 case records, we show here the daily evolution of confirmed new cases at the national level across three distinct data sources. First, we aggregate our subnational records to the national level; these time series are shown in purple. Second, we show in red national time series from the publicly available JHU Center for Systems Science and Engineering (JHU) data. Finally, in green we show national time series for European countries provided by the European Center for Disease Prevention and Control (ECDC). In most cases, the largest difference across datasets is due to the adjustments in dates we have made to accurately associate new cases to the date of their detection (Section B).

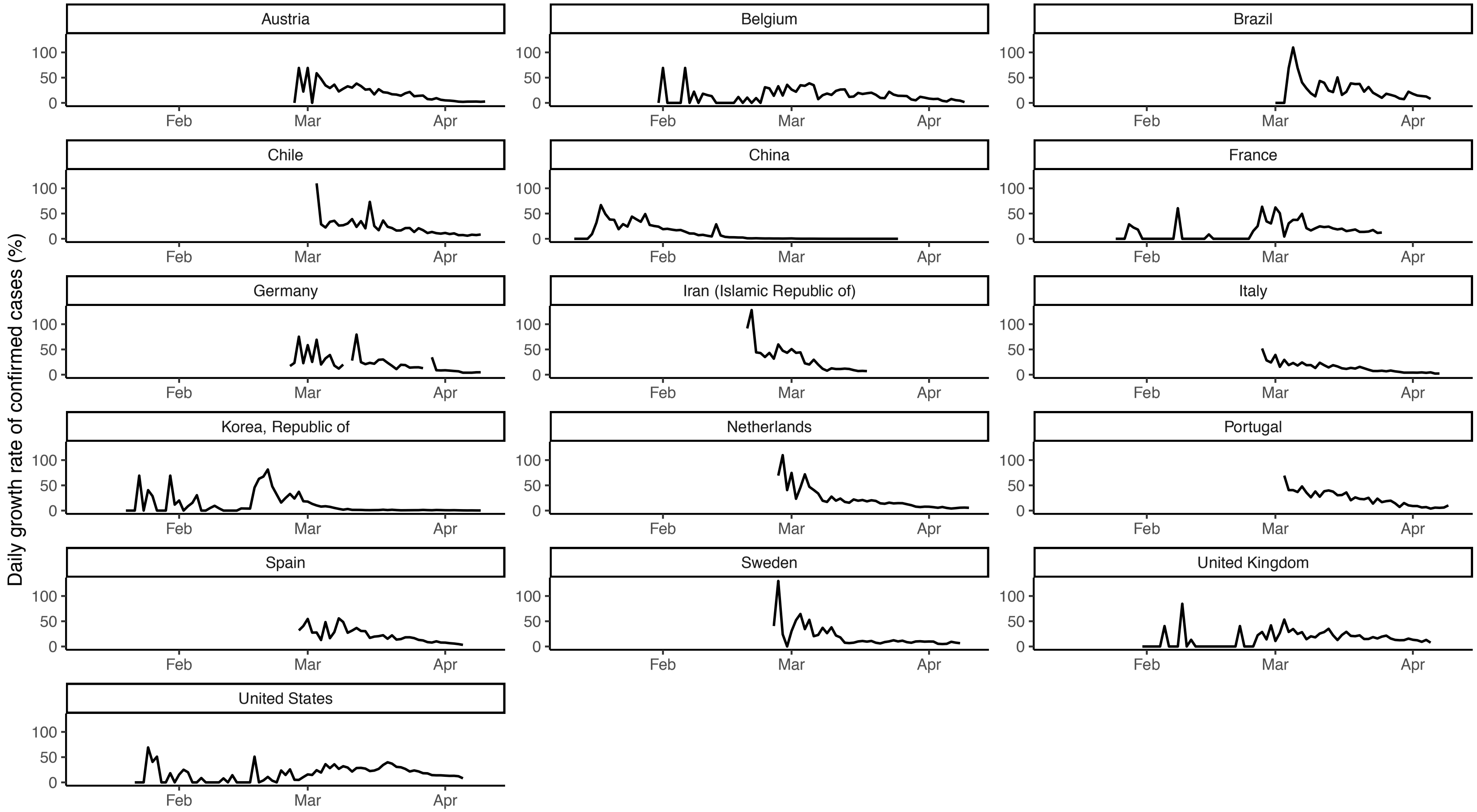

Figure S2: COVID-19 case growth rates in regions with subnational data. Here we aggregate confirmed subnational case data to the national level and plot the national COVID-19 growth rate over time from the regions where we have subnational data; subnational data from these regions comprise the majority of the sample observations used to estimate Eq. S1. Note that two missing values exist in Germany due to negative new case values, which we also drop from the analysis. Note further that in the analysis we use the subnational growth rates directly without aggregating.

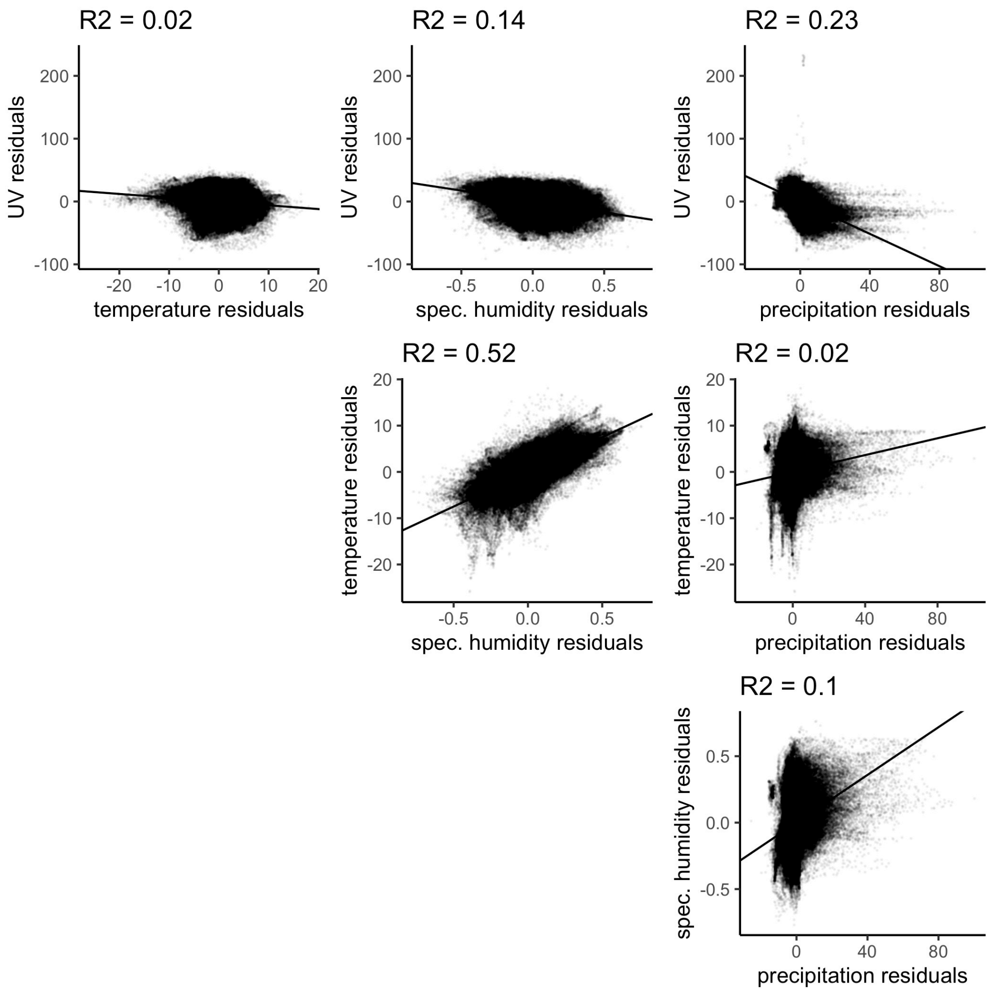

Figure S3: Correlation between daily environmental variables accounting for semi-parametric controls. Correlation between daily average UV (kJ/(m^2 hour)), average temperature (°C) , average humidity (%) and total precipitation (mm), after removing the semi-parametric controls in Eq. S1 (described in Section A.2). Linear fits are shown, with associated R^2 values.

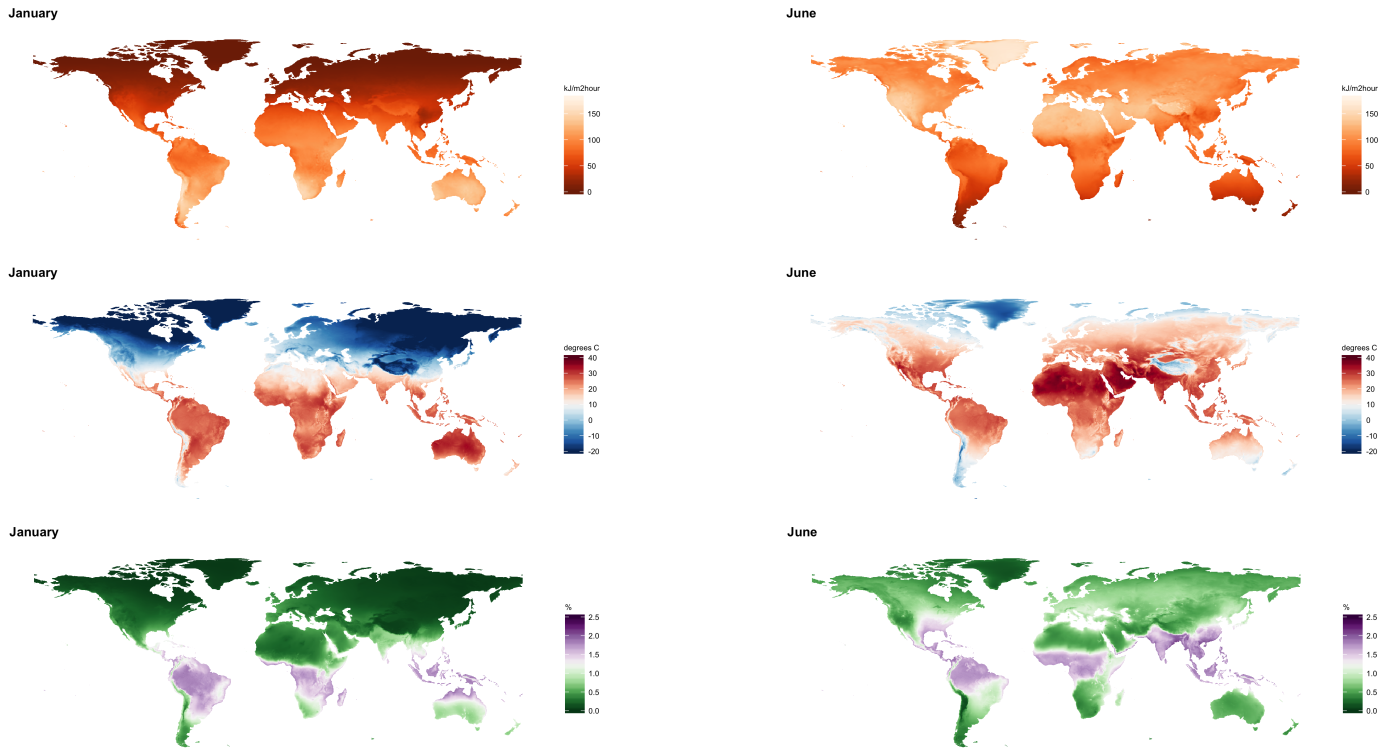

Figure S4: Seasonal changes in UV, temperature and specific humidity. Values show monthly averages over 2015-2019 for UV (kJ/m^2 hour), temperature (°C) and specific humidity (%) (across rows) and across January and June (across columns) at a 0.25° latitude by 0.25° longitude resolution. Raw hourly data are from the ERA5 reanalysis product produced by the European Centre for Medium-Range Weather Forecasts (ECMWF).

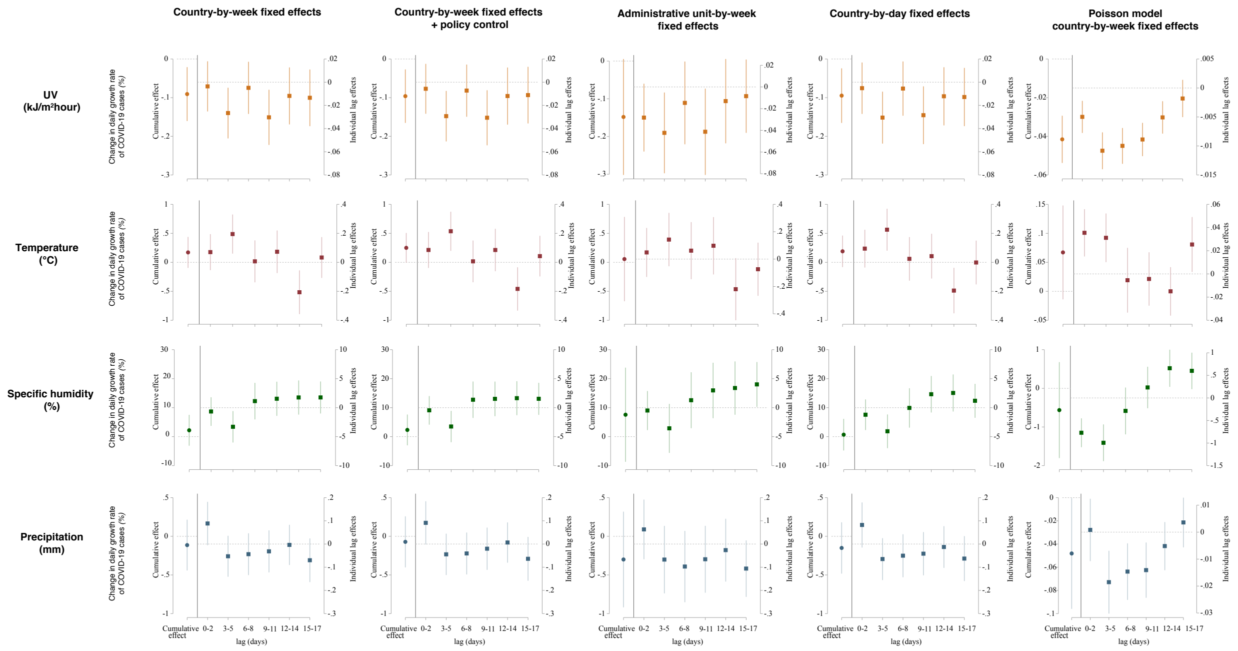

Figure S5: Alternative model specifications for the empirical relationship between daily growth rates of COVID-19 and climatological variables. Each column shows the estimated cumulative effect and dynamic response of the daily growth rate in COVID-19 cases to UV (gold), temperature (maroon), specific humidity (green), and precipitation (blue) under a distinct set of semi-parametric controls. All columns include administrative unit (e.g. country, province, county) "fixed effects" (i.e. dummies), and day of year fixed effects and all responses in each column are estimated jointly in a single regression. Column 1 includes country by week of year fixed effects and is our primary specification shown throughout the main text; column 2 includes country by week fixed effects and spatially and temporally-varying controls for social distancing policies (Section B.3); column 3 includes administrative unit (i.e. subnational units when available) by week fixed effects; column 4 drops all national-level data and adds county-by-day-of-year fixed effects. Finally, column 5 estimates a Poisson regression in which new cases per 1 million people are estimated as an exponential function of lagged climate variables, controlling for lagged total cumulative cases (Section A.2).

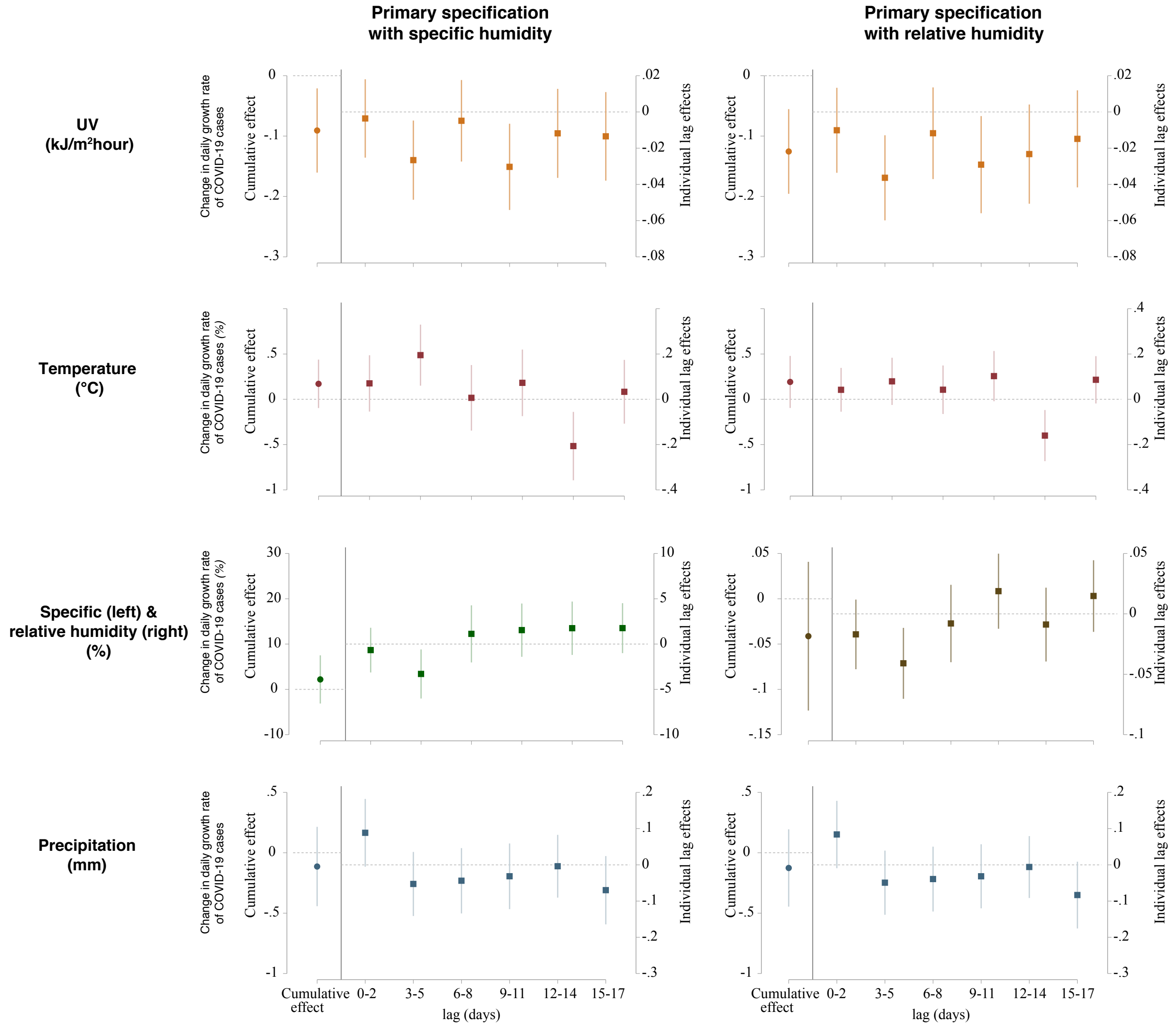

Figure S6: Empirical estimates of the dynamic relationship between COVID-19 and local climatic conditions using specific and relative humidity. Each column of this figure shows the estimated cumulative effect and dynamic response of the daily growth rate in confirmed COVID-19 cases to lagged 3-day average UV (gold), temperature (maroon), specific humidity (green), relative humidity (brown), and precipitation (blue). The left column shows our baseline specification (Fig. 3A,B). The right column is identical, except that the model is estimated using relative humidity (%) instead of specific humidity (kg/kg×100, or %). Note that these percentages represent conceptually different quantities. Relative humidity is the ratio of the partial pressure of water to the equilibrium vapor pressure, multiplied by 100, at a given temperature (i.e. what percent "full" of water is the air). Specific humidity gives the percent of an air parcel's total mass that is composed of water. The mean of relative humidity in our sample is 70% and the mean of specific humidity is 0.50%, which suggests that their estimated influences on the COVID-19 growth rate (i.e. the product of the estimated coefficients and changes in humidity) are of roughly similar magnitude.

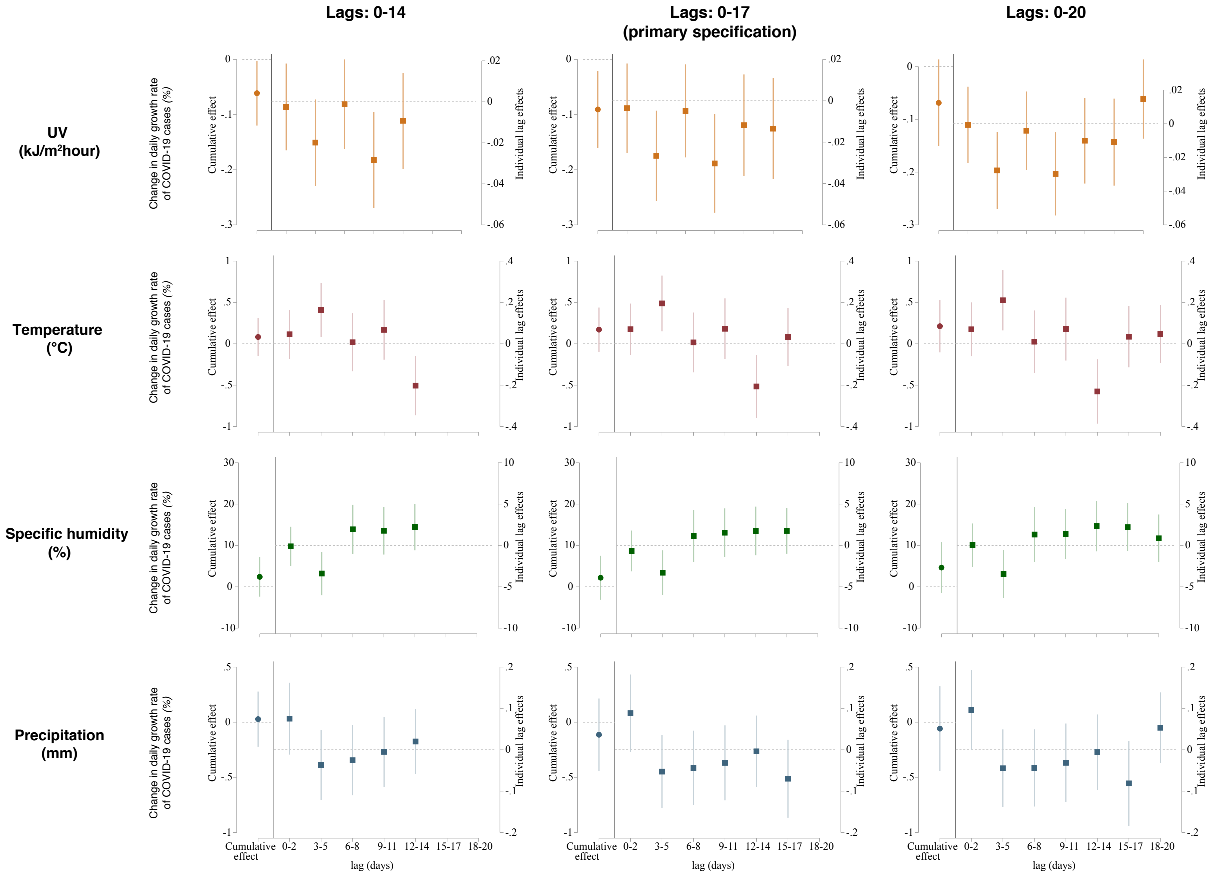

Figure S7: Empirical estimates of the dynamic relationship between COVID-19 and local climatic conditions using different distributed lag lengths. Each column of this figure shows the estimated cumulative effect and dynamic response of the daily growth rate in confirmed COVID-19 cases to lagged 3-day average UV (gold), temperature (maroon), specific humidity (green), and precipitation (blue) occurring up to 20 days prior. All coefficients in each column were estimated jointly in a statistical model leveraging a rich set of semi-parametric controls to isolate idiosyncratic variation in each weather variable (Section A.2). Point estimates are indicated by circles and 95% confidence intervals are indicated by vertical lines. The first row omits the 15-17 day lag, which is included in our baseline specification (Fig. 3A,B), the second row replicates our baseline specification, and the third row adds an additional 18-20 day lag.

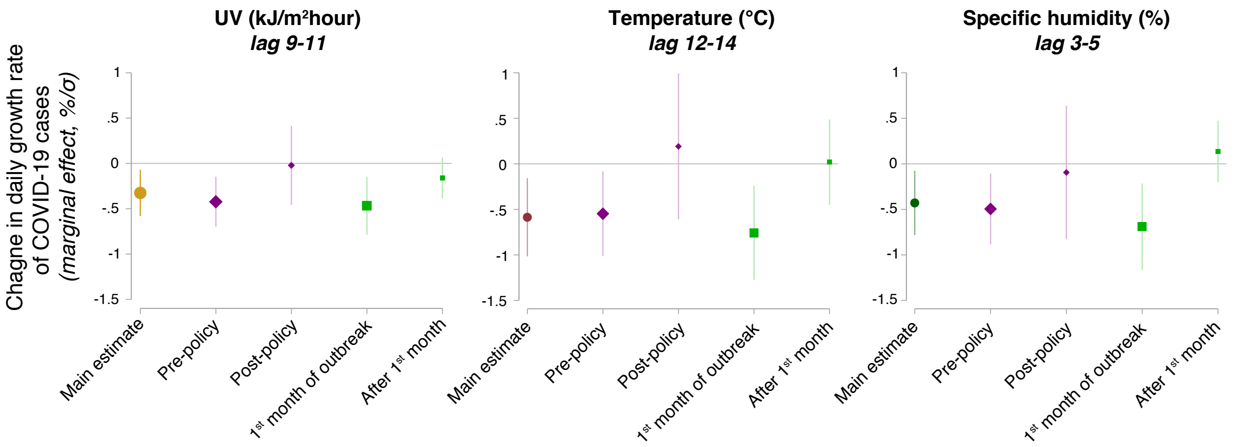

Figure S8: Individual lagged effects of climatological variables on daily growth rates of COVID-19 under distinct policy regimes and at different points in the outbreak. This figure shows the effect of select individual lag coefficients for each weather variable, with columns corresponding to UV (first column), temperature (second column), and specific humidity (third column). The lag shown for each climate variable is the largest magnitude effect recovered from a jointly estimated distributed lag regression including up to 17 days of lags (see Fig. S5, col. 1). In gold (UV), maroon (temperature), and dark green (specific humidity), the baseline specification used throughout the main text is shown. In purple, treatment effects of each weather variable are reported for the period of time before an administrative unit imposed any social distancing measures (large purple diamond), and after such measures were put in place (small purple diamond). Similarly, in light green, treatment effects of each weather variable are reported for the first 30 days of the location-specific outbreak (large green square), and for all dates after the first 30 days (small green square). Vertical lines indicate 95% confidence intervals. Effects of social distancing policies and outbreak duration on the cumulative effect of all lagged coefficients for all three weather variables are shown in Fig. 3C.

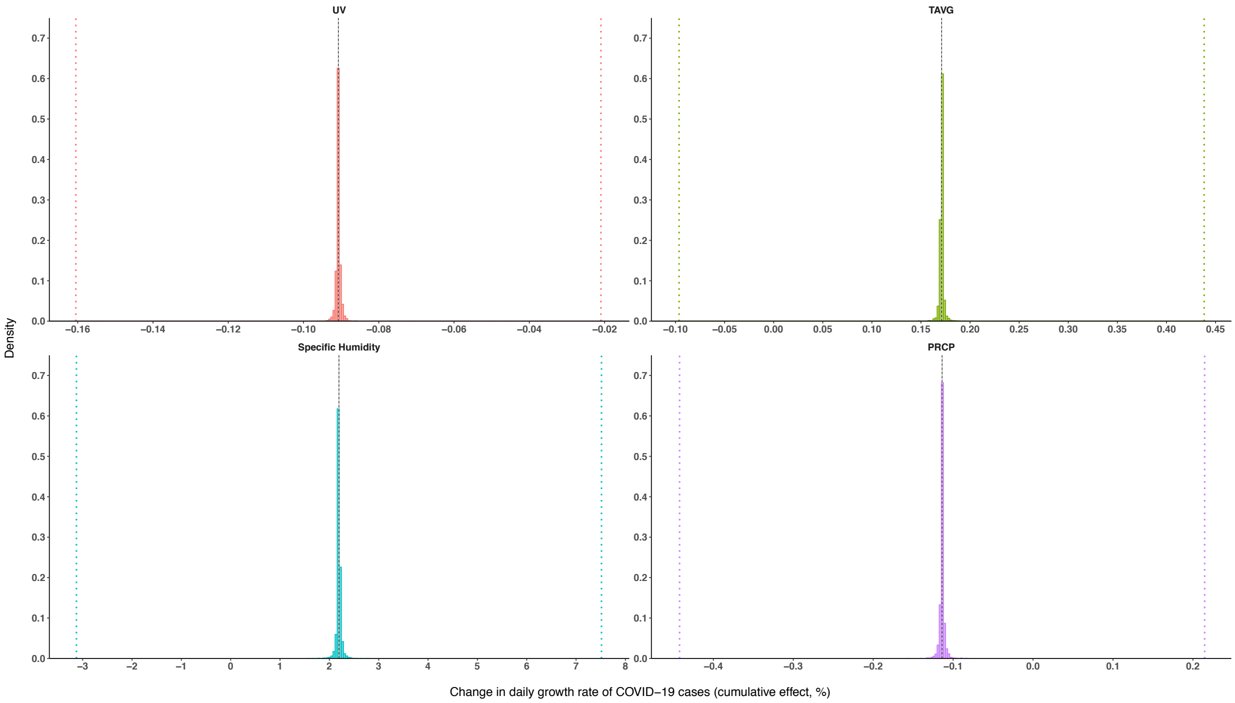

Figure S9: Estimates of cumulative effects of environmental variables on daily COVID-19 growth rates are insensitive to outliers. Daily growth rates in confirmed COVID-19 cases can be highly variable (Fig. 2A). To ensure that our primary estimates are not overly influenced by individual geospatial units with high variance, here we show the results of a block jackknife sensitivity analysis in which the entire time series corresponding to each of 3,235 geospatial units is removed from the dataset, and the primary estimating equation (Eq. S1) is re-run. Each subfigure shows a histogram of the cumulative effect (over 2.5 weeks, as reported in Fig. 3A) of UV, temperature, specific humidity, or precipitation, estimated using each of these 3,235 samples. Point estimates and corresponding 95% confidence intervals as computed in the main text using the full dataset (Fig. 3A) are shown with vertical dotted lines, demonstrating that primary estimates reported in the main text are robust to possible outliers.

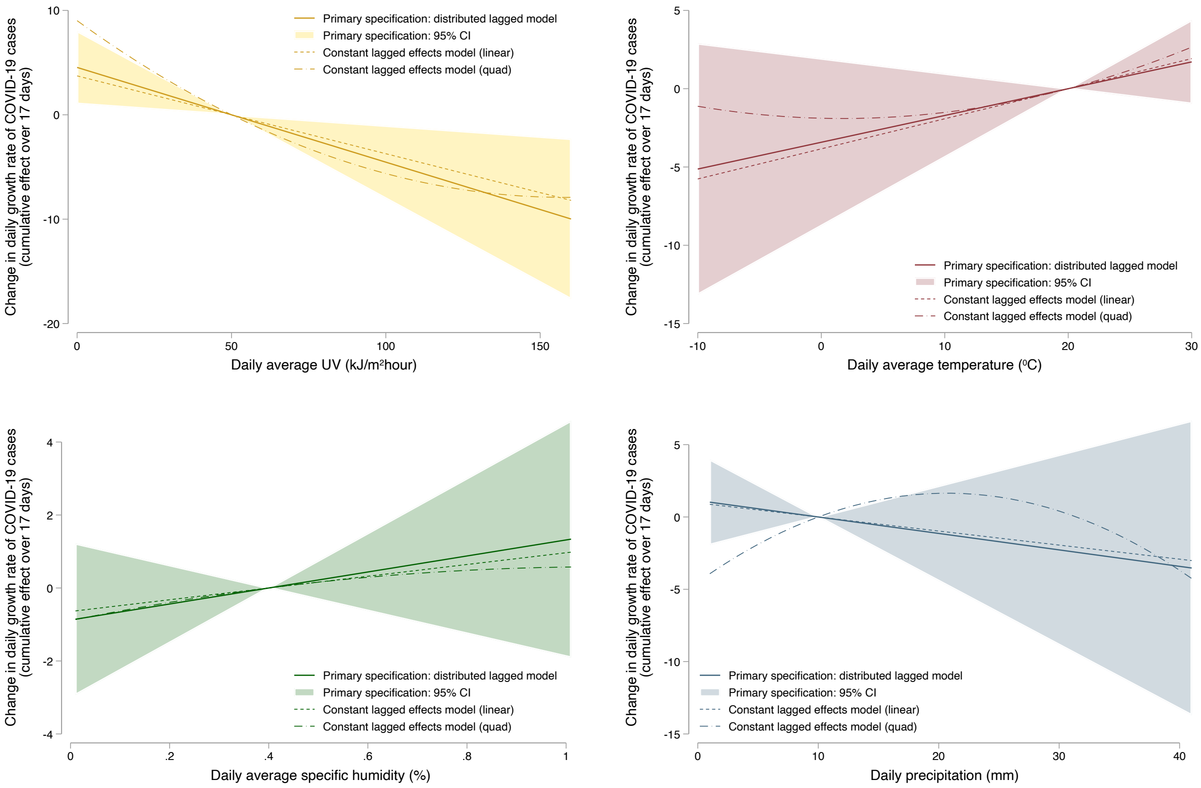

Figure S10: Potential nonlinear relationships between weather and change in the daily growth rate of COVID-19 cases, cumulative over 17 days. The solid line and 95% confidence interval plots shows the estimated cumulative effect from the temporal-distributed lag model in Eq. S1. The dashed line shows the analogous cumulative effect using the constant linear effects model from Eq. S3. The dotted-dashed line shows the analogous cumulative effect using the constant quadratic effects model in Eq. S4.

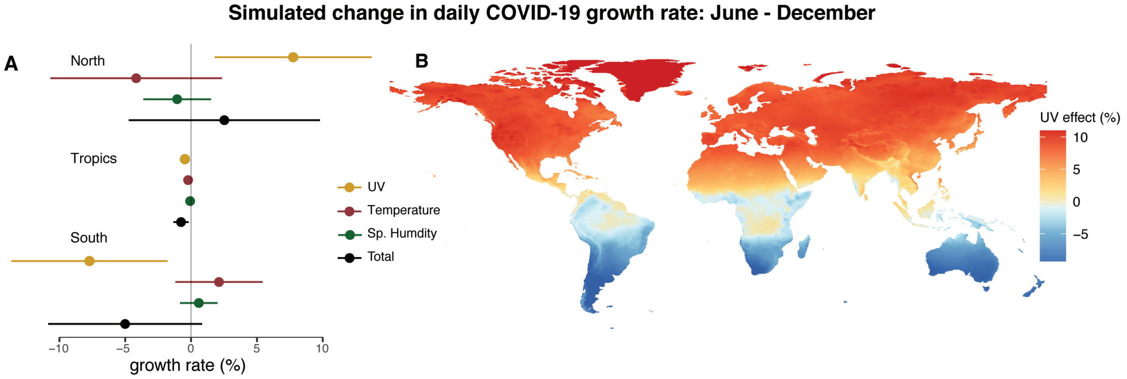

Figure S11: Seasonality in the simulated COVID-19 growth rate. Panel **A** shows the individual impacts of seasonal changes in UV (gold), temperature (maroon), and specific humidity (green), as well as their combined effect (black), from June to December. Points indicate average simulated impacts for northern latitudes, the tropics (23° south to 23° north), and southern latitudes. Horizontal lines show 95% confidence intervals, which account for uncertainty in statistical parameters. Panel **B** maps the influence of expected seasonal changes in UV alone on the COVID-19 growth rate from June to December.

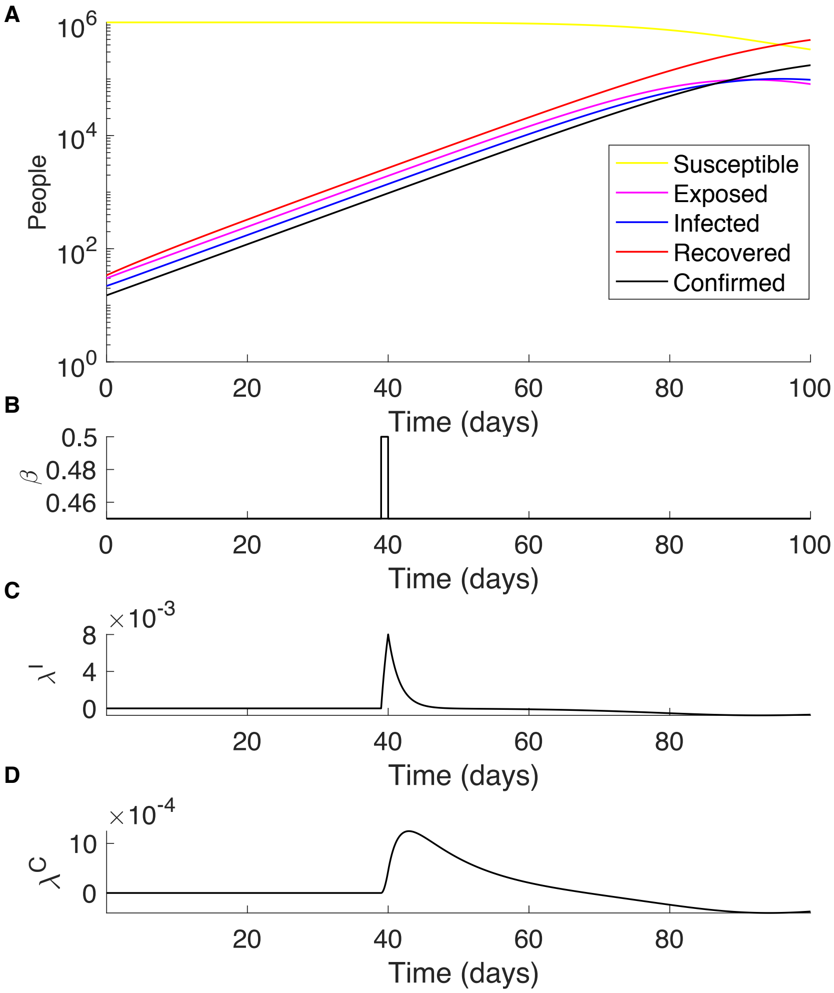

Figure S12: Simulated idealized dynamic response of the growth rate of infectious and confirmed cases to perturbed transmission in a deterministic SEIR model. We simulate the evolution of COVID-19 in a SEIR model deterministically using the semi-implicit Euler method (**A**, Section A.1). We let transmission, β, vary over time with linear disturbances due to changes in weather. In this idealized case, we generate a table-top perturbation in the weather, equal to zero except for a single day equaling one. In turn, this generates a day-long increase in β (**B**), which creates lagged increases in the growth rate of infected, λ^I, and growth rate of confirmed, λ^C, people, relative to a control run with constant β (**C**, **D**). This lagged response of λ^C to a single day change in weather is what we seek to capture in the COVID-19 confirmed case data using a statistical model.

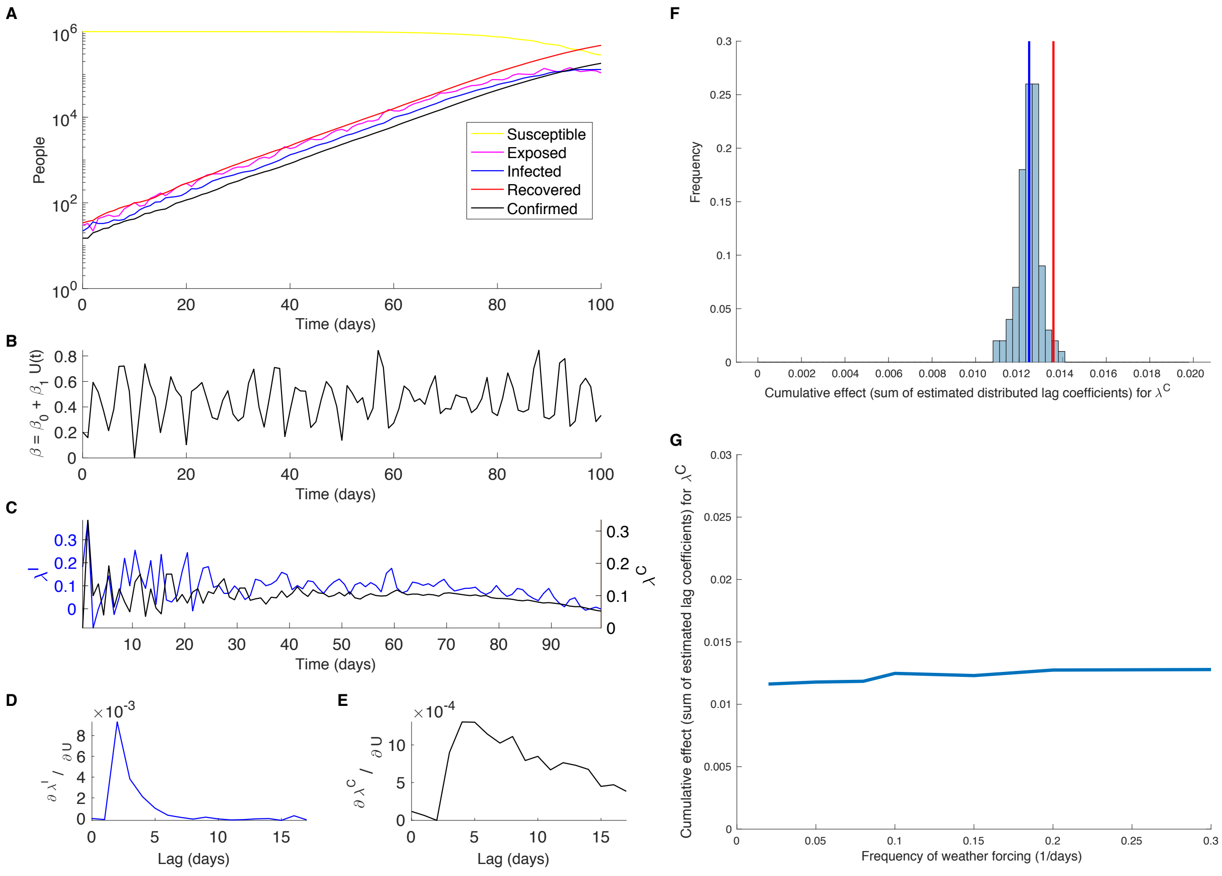

Figure S13: Recovering the dynamic influence of changes in transmission on the case growth rate by applying a temporal distributed lag regression model to simulated data from a stochastic SEIR model with time-varying transmission. We simulate the evolution of COVID-19 stochastically using a SEIR model (**A**, Section A.1). We let the weather forcing, U, which has a linear influence on transmission, β, be the sum of a sinusoid in t and Gaussian noise (**B**). We examine how a series of weather-induced time-varying shocks to transmission impact the growth rate of infectious and confirmed populations, λ^I and λ^C (**C**). Pooling observations from an ensemble of 500 runs, we estimate the effect of contemporaneous and lagged weather on λ^I and λ^C using a distributed lag regression model and recover a lagged response of these growth rates to weather-induced changes in transmission (**D**, **E**) similar in structure and magnitude to the idealized response from the deterministic experiment (Fig. S12C,D). Panel **F** shows the distribution of estimated cumulative effects for λ^C, which are the sum of lag coefficients like those shown in **E**, from 100 regression models trained on synthetic data. The vertical blue line shows the mean of these estimated cumulative effects. The red line shows the cumulative effect simulated in an idealized pulse experiment using a deterministic model (Fig. S12D). The agreement between the cumulative effect estimated by the regression model and that in the idealized pulse experiment (error $< 8\%$) motivates our application of the temporal lag regression model to COVID-19 data. Panel **G** shows the cumulative effect for λ^C estimated on data simulated using differing frequencies of weather forcing.

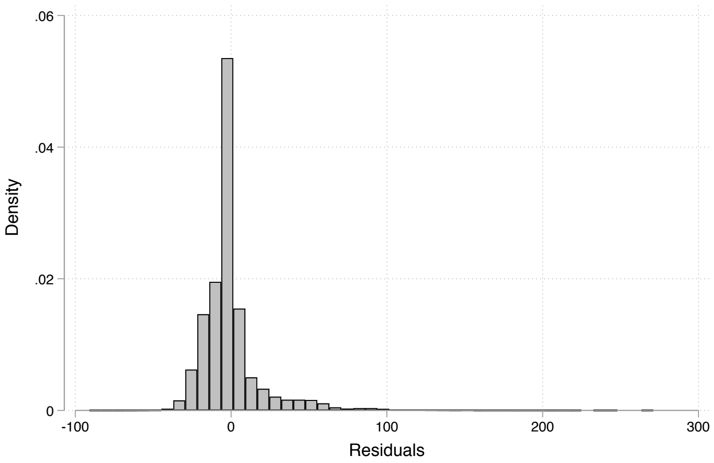

Figure S14: Distribution of residuals in daily growth rates. We estimate a distributed lag regression model in which the outcome variable is the growth rate in cumulative COVID-19 cases (Eq. S1). Here, we show the distribution of residuals after estimation of Eq. S1 using our baseline specification, which includes administrative unit fixed effects (i.e. dummy variables), country by week of year fixed effects, and day of year fixed effects (regression results shown in col. 3 of Table S1).

D Supporting Tables

	(1) OLS	(2) OLS	(3) OLS	(4) OLS	(5) OLS	(6) OLS	(7) OLS	(8) OLS	(9) Poisson
UV kJ/m2hour (lead: 1-3)				0.011					
				(0.012)					
UV kJ/m^2hour (lag: 0-2)	0.003	0.004	-0.004	-0.000	-0.006	-0.003	-0.028*	-0.005	-0.005***
	(0.011)	(0.011)	(0.011)	(0.012)	(0.011)	(0.011)	(0.016)	(0.011)	(0.001)
UV kJ/m^2hour (lag: 3-5)	-0.025**	-0.017	-0.027**	-0.025**	-0.029***	-0.027**	-0.042**	-0.031***	-0.011***
	(0.012)	(0.011)	(0.011)	(0.012)	(0.011)	(0.011)	(0.019)	(0.011)	(0.002)
UV kJ/m^2hour (lag: 6-8)	-0.003	0.005	-0.005	-0.005	-0.007	-0.005	-0.015	-0.006	-0.010***
	(0.012)	(0.011)	(0.011)	(0.013)	(0.011)	(0.011)	(0.019)	(0.012)	(0.002)
UV kJ/m^2hour (lag: 9-11)	-0.028**	-0.025**	-0.030**	-0.026*	-0.031**	-0.030**	-0.041**	-0.029**	-0.009***
	(0.013)	(0.012)	(0.012)	(0.014)	(0.012)	(0.012)	(0.020)	(0.013)	(0.001)
UV kJ/m^2hour (lag: 12-14)	-0.009	-0.011	-0.012	-0.003	-0.012	-0.011	-0.013	-0.012	-0.005***
	(0.013)	(0.013)	(0.013)	(0.014)	(0.013)	(0.013)	(0.020)	(0.013)	(0.001)
UV kJ/m^2hour (lag: 15-17)	-0.009	-0.006	-0.013	-0.017	-0.011	-0.013	-0.009	-0.013	-0.002
	(0.013)	(0.012)	(0.012)	(0.014)	(0.012)	(0.012)	(0.017)	(0.013)	(0.002)
Temp. °C (lead: 1-3)				0.043					
				(0.075)					
Temp. °C (lag: 0-2)	-0.016	0.030	0.070	0.055	0.085	0.069	0.048	0.094	0.035***
	(0.066)	(0.063)	(0.063)	(0.079)	(0.063)	(0.064)	(0.091)	(0.066)	(0.010)
Temp. °C (lag: 3-5)	0.119*	0.180***	0.195***	0.163**	0.214***	0.191***	0.143	0.225***	0.031***
	(0.070)	(0.069)	(0.069)	(0.076)	(0.069)	(0.069)	(0.099)	(0.074)	(0.011)
Temp. °C (lag: 6-8)	-0.041	0.035	0.007	0.041	0.006	0.008	0.062	0.024	-0.005
	(0.074)	(0.072)	(0.074)	(0.083)	(0.073)	(0.074)	(0.106)	(0.077)	(0.014)
Temp. °C (lag: 9-11)	0.015	0.063	0.073	0.036	0.085	0.073	0.097	0.043	-0.004
	(0.078)	(0.074)	(0.075)	(0.084)	(0.075)	(0.075)	(0.107)	(0.079)	(0.012)
Temp. °C (lag: 12-14)	-0.189**	-0.188**	-0.207***	-0.172**	-0.184**	-0.207***	-0.220*	-0.195**	-0.015
	(0.078)	(0.075)	(0.077)	(0.083)	(0.076)	(0.077)	(0.116)	(0.080)	(0.011)
Temp. °C (lag: 15-17)	0.044	-0.002	0.033	0.056	0.043	0.031	-0.074	-0.001	0.025**
	(0.071)	(0.070)	(0.072)	(0.076)	(0.071)	(0.072)	(0.099)	(0.077)	(0.012)
Humd. % (lead: 1-3)				-1.353					
				(1.427)					
Humd. % (lag: 0-2)	0.888	0.961	-0.668	0.826	-0.465	-0.756	-0.501	-1.222	-0.772***
	(1.285)	(1.206)	(1.259)	(1.515)	(1.259)	(1.262)	(1.720)	(1.347)	(0.163)
Humd. % (lag: 3-5)	-1.661	-3.417**	-3.302**	-2.488	-3.256**	-3.229**	-3.568*	-4.077***	-0.993***
	(1.417)	(1.358)	(1.382)	(1.606)	(1.379)	(1.386)	(2.158)	(1.483)	(0.208)
Humd. % (lag: 6-8)	2.325	-0.690	1.124	1.544	1.367	1.043	1.284	-0.049	-0.288
	(1.627)	(1.579)	(1.609)	(1.791)	(1.601)	(1.612)	(2.462)	(1.734)	(0.265)
Humd. % (lag: 9-11)	2.160	-0.136	1.536	1.862	1.515	1.600	2.969	2.315	0.232
	(1.531)	(1.477)	(1.501)	(1.593)	(1.523)	(1.507)	(2.441)	(1.607)	(0.235)
Humd. % (lag: 12-14)	0.734	0.520	1.749	1.822	1.634	1.713	3.378	2.533	0.658***
	(1.459)	(1.457)	(1.503)	(1.610)	(1.494)	(1.507)	(2.347)	(1.631)	(0.210)
Humd. % (lag: 15-17)	0.158	1.609	1.756	0.531	1.526	1.773	4.006**	1.183	0.600***
	(1.373)	(1.378)	(1.407)	(1.532)	(1.410)	(1.411)	(1.974)	(1.493)	(0.206)
UV kJ/m^2hour (cum. effect)	-.071*	-.051	-.091**	-.075**	-.096***	-.089**	-.148*	-.095***	-.042***
	(.042)	(.037)	(.036)	(.037)	(.035)	(.036)	(.078)	(.036)	(.006)
Temp. °C (cum. effect)	-.068	.118	.171	.179	.25*	.164	.056	.19	.067
	(.168)	(.138)	(.136)	(.161)	(.131)	(.136)	(.371)	(.139)	(.041)
Humd. % (cum. effect)	4.604	-1.154	2.194	4.097	2.322	2.144	7.566	.683	-.563
	(3.227)	(2.737)	(2.715)	(3.113)	(2.71)	(2.717)	(8.286)	(2.782)	(.634)
Observations	51139	51140	51126	43626	51126	50740	48328	48813	45292
R-squared	.21	.22	.22	.25	.22	.22	.33	.23	.74
Day FE	Yes	Yes	Yes	Yes	Yes	Yes	Yes	Yes	Yes
Days since outbk. ctl.	Yes	No	No	No	No	No	No	No	No
Country trend	No	Yes	No	No	No	No	No	No	No
CntryXweek FE	No	No	Yes	Yes	Yes	Yes	No	No	Yes
Policy ctl.	No	No	No	No	Yes	No	No	No	No
Testing ctl.	No	No	No	No	No	Yes	No	No	No
AdminXweek FE	No	No	No	No	No	No	Yes	No	No
CntryXday FE	No	No	No	No	No	No	No	Yes	No

Table S1: Empirical estimation of the relationship between COVID-19 and climatological variables. Columns (1)-(8) show estimates of the distributed lag regression model from Eq. (S1) using daily longitudinal data across a pooled sample of national and subnational data (Fig. 1). The outcome is the daily growth rate of cumulative confirmed cases for columns (1) through (8). In column (9), a Poisson distributed lag regression model is used (Eq. S2). All models include administrative unit (e.g. country, province, or county) and day of year fixed effects, and all control for distributed lags in daily precipitation (in mm) and specific humidity (in %). Columns (1)-(8) include distinct semi-parametric and other controls: (1) a "fixed effect" (dummy variable) for the number of days since the outbreak began; (2) linear country-specific time trend; (3) country by week fixed effects; (4) country by week fixed effects, including leads of climate variables; (5) country by week fixed effects, including controls for temporally and spatially-varying social distancing policy controls (Section B.3); (6) country by week fixed effects, including a control for the stringency of COVID-19 testing at country level (Section B); (7) administrative unit (e.g. country, province, county) by week fixed effects; (8) country by day fixed effects. Standard errors clustered at the administrative unit level are in parentheses. P-values from two-sided t-tests with *** $p<0.01$, ** $p<0.05$, * $p<0.1$.

Country	Resolution	Units	Time coverage	Source	Variable obtained	Note
Austria	Bundesland	9	2/26 - 4/8	Ministry of Health[1]	Cumulative cases	
Belgium	Region	3	1/30 - 4/6	Public Health Institute[2]	New cases	
Brazil	State	27	2/25 - 4/5	Ministry of Health[3]	New cases	
Chile	Region	16	3/2 - 4/6	Ministry of Health[4]	New cases	Date corrected
China	City	339	1/10 - 3/25	Health Professionals network[5]	Cumulative cases	Imputed missing values
France	Region	13	1/24 - 3/25	Various[6]	New cases	
Germany	Land	16	2/24 - 4/7	Public Health Institute[7]	Cumulative cases	Date corrected; Missing values
Iran	Province	31	2/19 - 3/22	Ministry of Health, News Agency[8]	New cases	Imputed mising values
Italy	Province	107	2/24 - 4/7	Department of Civil Protection[9]	Cumulative cases	
Netherlands	Province	12	2/26 - 4/7	Public Health Institute [10]	New cases	Date corrected
Portugal	Region	7	3/2 - 4/7	Directorate-General of Health[11]	New cases	Date corrected
South Korea	Province	17	1/19 - 4/9	Public Health Institute[12]	New cases	Date corrected
Spain	A. C.	16	2/26 - 4/5	Ministry of Health[13]	Cumulative cases	Date corrected
Sweden	County	21	2/25 - 4/8	Public Health Agency[14]	New cases	Date corrected
United Kingdom	Country	4	1/30 - 4/5	Public Health Institute[15]	New cases	Date corrected
United States	County	2,438	1/21 - 4/5	Various[16]	Cumulative cases	

Table S2: COVID-19 cases data for subnational units. This table contains information about the COVID-19 cases database. It presents the time and spatial coverage of our subnational data, the sources and the variables provided by these sources. It also indicates the corrections we applied to the data.

[1] *Bundesministerium für Soziales, Gesundheit, Pflege und Konsumentschutz*, https://www.sozialministerium.at/Informationen-zum-Coronavirus/Neuartige-Coronavirus-(2019-nCov).html; scraped from the Wikipedia article "COVID-19-Pandemie in Österreich" ("COVID-19 pandemic in Austria"), available at https://de.wikipedia.org/wiki/COVID-19-Pandemie_in_%C3%96sterreich.

[2] *Sciensano*, https://epistat.wiv-isp.be/covid/, obtained from the Wikipedia article "2020 coronavirus pandemic in Belgium", https://en.wikipedia.org/wiki/2020_coronavirus_pandemic_in_Belgium

[3] *Ministério da Saúde*, https://covid.saude.gov.br/, obtained from the GitHub repository of Henrico Moraes, https://github.com/elhenrico/covid19-Brazil-timeseries

[4] *Ministerio de Salud*, https://www.minsal.cl/nuevo-coronavirus-2019-ncov/casos-confirmados-en-chile-covid-19/; scraped from the Wikipedia article "2020 coronavirus pandemic in Chile" (https://en.wikipedia.org/w/index.php?title=2020_coronavirus_pandemic_in_Chile).

[5] Dīng Xiāng Yuán (DXY), https://ncov.dxy.cn/ncovh5/view/en_pneumonia?from=dxy&source=&link=&share=; obtained from the Github repository of the Global Policy Lab: https://github.com/bolliger32/gpl-covid

[6] The compiled data have been scraped from the Wikipedia article"2020 coronavirus pandemic in France", https://en.wikipedia.org/wiki/2020_coronavirus_pandemic_in_France.

[7] Robert Koch Institute, https://www.rki.de/DE/Content/InfAZ/N/Neuartiges_Coronavirus/Fallzahlen.html. The data have been scraped from the Wikipedia article "2020 coronavirus pandemic in Germany", available at https://en.wikipedia.org/wiki/2020_coronavirus_pandemic_in_Germany.

[8] https://en.irna.ir/photo/83723991/Iran-s-coronavirus-toll-update-March-22-2020. The compiled data have been scraped from the Wikipedia article "2020 coronavirus pandemic in Iran", available at https://en.wikipedia.org/wiki/2020_coronavirus_pandemic_in_Iran.

[9] *Dipartimento della Protezione Civile*, http://opendatadpc.maps.arcgis.com/apps/opsdashboard/index.html#/b0c68bce2cce478eaac82fe38d4138b1. The data are directly available on their GitHub repository, at https://github.com/pcm-dpc/COVID-19.

[10] Institute for Public Health and the Environment, https://www.rivm.nl/en/novel-coronavirus-covid-19/current-information-about-novel-coronavirus-covid-19. The data are available on the Wikipedia page "2019-20 coronavirus pandemic data, Netherlands medical cases", https://en.wikipedia.org/wiki/Template:2019%E2%80%9320_coronavirus_pandemic_data/Netherlands_medical_cases.

[11] *Direção-Geral da Saúde*, https://covid19.min-saude.pt/relatorio-de-situacao/. The data have been scraped from the Wikipedia article "2020 coronavirus pandemic in Portugal" (https://en.wikipedia.org/wiki/2020_coronavirus_pandemic_in_Portugal).

[12] Korean Center for Disease Control and Prevention, https://www.cdc.go.kr/board/board.es?mid=a30402000000&bid=0030. The data have been obtained from the Wikipedia article "2020 coronavirus pandemic in South Korea", available at https://en.wikipedia.org/wiki/2020_coronavirus_pandemic_in_South_Korea.

[13] *Ministerio de Sanidad – Centro de Coordinación de Alertas y Emergencias Sanitarias*, https://www.mscbs.gob.es/profesionales/saludPublica/ccayes/alertasActual/nCov-China/situacionActual.htm. The data have been compiled by Datadista and made available on their GitHub repository, at https://github.com/datadista/datasets/tree/master/COVID%2019.

[14] *Folkhälsomyndigheten*, https://experience.arcgis.com/experience/09f821667ce64bf7be6f9f87457ed9aa. The data have been compiled from the Wikipedia article "2020 coronavirus pandemic in Sweden", (https://en.wikipedia.org/wiki/2020_coronavirus_pandemic_in_Sweden).

[15] Public Health England, https://www.arcgis.com/apps/opsdashboard/index.html#/f94c3c90da5b4e9f9a0b19484dd4bb14. The data have been scraped from the Wikipedia article "2020 coronavirus pandemic in the United Kingdom", available at https://en.wikipedia.org/wiki/2020_coronavirus_pandemic_in_the_United_Kingdom.

[16] All data have been meticulously compiled by the *New York Times*, and can be downloaded from the GitHub repository they dedicated to this issue: https://github.com/nytimes/covid-19-data/archive/master.zip.

Country	Resolution	Year	Source
Austria	Bundesland	January 2015	Statistik Austria[1]
Belgium	Region	January 2015	STATBEL[2]
Brazil	State	July 2019	Brazilian Institute of Geography and Statistics (IBGE)[3]
Chile	Region	2017	Instituto Nacional de Estadísticas (INE)[4]
China	City	2010	National Bureau of Statistics of China[5]
France	Region	January 2017	INSEE[6]
Germany	Land	December 2018	Statistisches Bundesamt[7]
Iran	Province	2016	Statistical Center of Iran[8]
Italy	Province	January 2019	ISTAT[9]
Netherlands	Province	November 2019	Statistics Netherlands (CBS)[10]
Portugal	Region	December 2018	Statistics Portugal (INE)[11]
South Korea	Province	December 2017	citypopulation.de[12]
Spain	Autonomous Community	2019	Spanish Statistical Office (INE)[13]
Sweden	County	December 2018	Statistics Sweden (SCB).[14]
United Kingdom	Country	2018	Office for National Statistics[15]
United States	County	July 2019	US Census Bureau[16]

Table S3: Population data at the subnational level. Our population data for each subnational unit come mostly from national offices of statistics, listed in this table. This table also indicates when the population estimates were made.

[1] Available at http://www.statistik.at/web_de/statistiken/menschen_und_gesellschaft/bevoelkerung/080912.html (in German). The data have been scraped from the Wikipedia article "States of Austria", available at https://en.wikipedia.org/w/index.php?title=States_of_Austria&oldid=947664334.

[2] Available at https://statbel.fgov.be/fr/themes/population/structure-de-la-population (in French).

[3] Available here: https://www.ibge.gov.br/en/statistics/social/population/18448-estimates-of-resident-population-for-municipalities-and-federation-units.html?=&t=resultados.

[4] Downloadable here: https://www.ine.cl/estadisticas/sociales/censos-de-poblacion-y-vivienda/poblacion-y-vivienda (in Spanish).

[5] http://www.stats.gov.cn/tjsj/ndsj/2010/indexeh.htm. The data have been downloaded from the GitHub repository of the Global Policy Lab, available at https://github.com/bolliger32/gpl-covid.

[6] Available here: https://www.insee.fr/fr/statistiques/4265390?sommaire=4265511 (in French). The data have been scraped from the Wikipedia article "Région française" and are available at https://fr.wikipedia.org/wiki/R%C3%A9gion_fran%C3%A7aise.

[7] Retrievable here: https://www-genesis.destatis.de/genesis/online?operation=abruftabelleBearbeiten&levelindex=2&levelid=1587183994407&auswahloperation=abruftabelleAuspraegungAuswaehlen&auswahlverzeichnis=ordnungsstruktur&auswahlziel=werteabruf&code=12411-0010&auswahltext=&werteabruf=Value+retrieval#astructure. The data have been scraped from the Wikipedia article "List of German States by Population" (https://en.wikipedia.org/wiki/List_of_German_states_by_population).

[8] https://www.amar.org.ir/english/Population-and-Housing-Censuses. The data have been scraped from the Wikipedia article "Provinces of Iran", available at https://en.wikipedia.org/wiki/Provinces_of_Iran.

[9] Available at http://demo.istat.it/pop2019/index3_e.html. The data have been downloaded from the GitHub repository of the Global Policy Lab, available at https://github.com/bolliger32/gpl-covid.

[10] Available here: https://opendata.cbs.nl/statline/#/CBS/nl/dataset/37230ned/table (in Dutch). The data have been scraped from the Wikipedia article "Provinces of the Netherlands", available at https://en.wikipedia.org/wiki/Provinces_of_the_Netherlands.

[11] Downloadable here: https://www.ine.pt/xportal/xmain?xpid=INE&xpgid=ine_publicacoes&PUBLICACOESpub_boui=411651997&PUBLICACOEStema=00&PUBLICACOESmodo=2.

[12] https://www.citypopulation.de/en/southkorea/cities/

[13] Available at https://www.ine.es/jaxiT3/Tabla.htm?t=4925&L=0 (in Spanish)

[14] The original data can be found at https://www.scb.se/en/finding-statistics/statistics-by-subject-area/population/population-composition/population-statistics/pong/tables-and-graphs/quarterly-population-statistics--municipalities-counties-and-the-whole-country/julydecember-2018/.

[15] Complete report available here: https://www.ons.gov.uk/peoplepopulationandcommunity/populationandmigration/populationestimates/bulletins/annualmidyearpopulationestimates/mid2018.

[16] Downloadable at: https://www2.census.gov/programs-surveys/popest/tables/2010-2019/counties/totals/co-est2019-annres.xlsx.

Supporting References

[1] Kissler, S. M., Tedijanto, C., Goldstein, E., Grad, Y. H. & Lipsitch, M. Projecting the transmission dynamics of SARS-CoV-2 through the postpandemic period. *Science* (2020).

[2] Anderson, R. M., Heesterbeek, H., Klinkenberg, D. & Hollingsworth, T. D. How will country-based mitigation measures influence the course of the covid-19 epidemic? *The Lancet* **395**, 931–934 (2020).

[3] Kogan, N. E. *et al.* An early warning approach to monitor covid-19 activity with multiple digital traces in near real-time. *arXiv preprint arXiv:2007.00756* (2020).

[4] Russell, T. W. *et al.* Estimating the infection and case fatality ratio for coronavirus disease (covid-19) using age-adjusted data from the outbreak on the diamond princess cruise ship, february 2020. *Eurosurveillance* **25**, 2000256 (2020).

[5] Britton, T. & Pardoux, E. *Stochastic Epidemic Models with Inference* (Springer, Cham, Switzerland, 2019).

[6] Ma, J. Estimating epidemic exponential growth rate and basic reproduction number. *Infectious Disease Modelling* **5**, 129–141 (2020).

[7] Lauer, S. A. *et al.* The incubation period of coronavirus disease 2019 (COVID-19) from publicly reported confirmed cases: Estimation and application. *Annals of Internal Medicine* (2020).

[8] Li, Q. *et al.* Early transmission dynamics in Wuhan, China, of novel coronavirus–infected pneumonia. *New England Journal of Medicine* (2020).

[9] Jiang, X., Rayner, S. & Luo, M.-H. Does sars-cov-2 has a longer incubation period than sars and mers? *Journal of medical virology* **92**, 476–478 (2020).

[10] Nie, X. *et al.* Epidemiological characteristics and incubation period of 7015 confirmed cases with coronavirus disease 2019 outside hubei province in china. *The Journal of Infectious Diseases* (2020).

[11] Deschênes, O. & Greenstone, M. The economic impacts of climate change: Evidence from agricultural output and random fluctuations in weather. *American Economic Review* **97**, 354–385 (2007).

[12] Hsiang, S. Climate econometrics. *Annual Review of Resource Economics* **8**, 43–75 (2016).

[13] Cameron, A. C. & Trivedi, P. K. *Regression analysis of count data*, vol. 53 (Cambridge university press, 2013).

[14] Hsiang, S. *et al.* The effect of large-scale anti-contagion policies on the COVID-19 pandemic. *Nature* .

[15] Dong, E., Du, H. & Gardner, L. An interactive web-based dashboard to track COVID-19 in real time. *The Lancet Infectious Diseases* (2020).

[16] Hale, T., Petherick, A., Phillips, T. & Webster, S. Variation in government responses to COVID-19: version 4.0. *Blavatnik School of Government Working Paper* (2020).

[17] Copernicus Climate Change Service. ERA5: Fifth generation of ECMWF atmospheric reanalyses of the global climate ("2017"). URL https://cds.climate.copernicus.eu/cdsapp#!/home. Date of access: 2020-03-20.

[18] Bright, E. A., P. R. Coleman, A. N. Rose, and M. L. Urban. LandScan 2011 ("2012"). URL web.ornl.gov/sci/landscan/index.shtml.
